# Immunomodulatory Tissue‐Engineering Strategies for Diabetic Foot Ulcer Management: A Systematic Review

**DOI:** 10.1111/wrr.70149

**Published:** 2026-03-25

**Authors:** Gözde Özsezer, Yavuz Emre Arslan

**Affiliations:** ^1^ Faculty of Health Sciences, Department of Public Health Nursing Çanakkale Onsekiz Mart University Çanakkale Turkey; ^2^ Regenerative Biomaterials Laboratory, Department of Bioengineering, Faculty of Engineering Çanakkale Onsekiz Mart University Çanakkale Turkey

**Keywords:** diabetic foot, immunomodulation, macrophages, tissue engineering, wound healing

## Abstract

The aim of this systematic review is to systematically compile evidence from the past 5 years on bioengineering and regenerative medicine approaches targeting the DFU immune microenvironment and to evaluate these findings from a translational perspective to inform clinical applications. This systematic review, conducted according to PRISMA 2020 guidelines, summarised evidence from 34 studies published between 2020 and 2025 on immunomodulatory tissue‐engineering strategies for DFU management. Most studies used STZ‐induced or db/db diabetic mouse models; only two included human data. Across natural polymer hydrogels, electrospun nanofibers, microneedles, and hybrid antimicrobial dressings, a consistent mechanistic theme emerged: promotion of macrophage polarisation from pro‐inflammatory M1 to pro‐regenerative M2. Cytokine delivery, exosome‐based therapies, ROS‐targeted nanozymes, metabolic reprogramming, and electrical or microcurrent stimulation resolved chronic inflammation, enhanced angiogenesis, and accelerated wound closure. Key signalling pathways (JAK/STAT, NF‐κB, HMGB1–RAGE, HIF‐1α) represent promising molecular targets. Despite encouraging preclinical outcomes, heterogeneity and limited human studies underscore the need for well‐powered, long‐term clinical trials and biomarker‐driven personalised immunomodulatory strategies.

**Abbreviations**
AAPBA: 3‐acrylamidophenylboronic acid
ADM: acellular dermal matrix
AGE(s): advanced glycation end products
ATMP: advanced therapy medicinal product
AUC: area under the receiver operating characteristic curve
bFGF: Basic fibroblast growth factor (FGF‐2)
BMDM: bone marrow–derived macrophage
CAT: catalase
CD31: platelet endothelial cell adhesion molecule‐1 (PECAM‐1)
CM: conditioned medium
db/db: leptin receptor–deficient diabetic mouse model
DDMNS: double‐layer detachable microneedle system
DFO: deferoxamine
DFU: diabetic foot ulcer
DM: diabetes mellitus
ECM: extracellular matrix
EGF: epidermal growth factor
EMA: European Medicines Agency
ER: endoplasmic reticulum
EV(s): extracellular vesicle(s)
FDA: U.S. Food and Drug Administration
FG: fish gelatin
FGMA: fish gelatin methacrylate
FOXM1: forkhead box protein M1
GBC: guanosine–phenylboronic acid–chlorogenic acid (hydrogel)
GelMA: gelatin methacrylate
GEO: gene expression omnibus
GMP: good manufacturing practice
GOx: glucose oxidase
HA: hyaluronic acid
HA‐AC: acrylated hyaluronic acid
HaCaT: Immortalised human keratinocyte cell line
HAMA: hyaluronic acid methacrylate
HAQ: HAMA–AAPBA + QCS capture‐and‐kill hydrogel
HIF‐1α: hypoxia‐inducible factor‐1 alpha
HMGB1: high‐mobility group box 1
HSE: human skin equivalent
HUVEC: human umbilical vein endothelial cell
IDF: International Diabetes Federation
IGF: insulin‐like growth factor
IL: interleukin
IL‐1R1: interleukin‐1 receptor type 1
IL‐1Ra: interleukin‐1 receptor antagonist
IL‐27Rα: interleukin‐27 receptor alpha
JAK/STAT: Janus kinase/signal transducer and activator of transcription
JBI: Joanna Briggs Institute
KDELR3: KDEL endoplasmic reticulum protein retention receptor 3
KEAP1: Kelch‐like ECH‐associated protein 1
LBD(s): Lactobacillus biofilm derivative(s)
M1/M2: classically activated (pro‐inflammatory)/alternatively activated (pro‐regenerative) macrophage phenotypes
MBG: mesoporous bioactive glass
MC3T3‐E1: Murine pre‐osteoblast cell line
MMP: matrix metalloproteinase
MnCoO@PDA: manganese–cobalt oxide @ polydopamine (nanozyme)
MSC: mesenchymal stem cell
NET: neutrophil extracellular trap
NF‐κB: nuclear factor kappa‐B
NIR: near‐infrared
NRF2: nuclear factor erythroid 2–related factor 2
OHAT: Office of Health Assessment and Translation
OI: 4‐octyl itaconate
PCL: poly(ε‐caprolactone)
PDA: polydopamine
PDGF: platelet‐derived growth factor
PdH: palladium hydride
PD‐L1: programmed death‐ligand 1
PEG: poly(ethylene glycol)
PI3K/Akt/mTOR: phosphoinositide 3‐kinase/protein kinase B/mechanistic target of rapamycin
PLGA: poly(lactic‐co‐glycolic acid)
PNS: Panax notoginseng saponins
Ppy: polypyrrole
PRISMA: Preferred Reporting Items for Systematic Reviews and Meta‐Analyses
PROSPERO: International Prospective Register of Systematic Reviews
PVA: poly(vinyl alcohol)
PVB: poly(vinyl butyral)
RAGE: receptor for advanced glycation end products
RCT: randomised controlled trial
rGO: reduced graphene oxide
ROS: reactive oxygen species
SDF‐1α: stromal cell–derived factor‐1 alpha (CXCL12)
SMAD: mothers against decapentaplegic homologue proteins
SOD: superoxide dismutase
STAT1/3, pSTAT3: signal transducer and activator of transcription 1/3; phosphorylated STAT3
STZ: streptozotocin
SYRCLE: Systematic Review Centre for Laboratory animal Experimentation
TGF‐β: transforming growth factor‐beta
TLR: toll‐like receptor
TREM1: triggering receptor expressed on myeloid cells 1
VEGF‐A: vascular endothelial growth factor‐A
WGCNA: weighted gene co‐expression network analysis
YOD1: YOD1 deubiquitinase
Zn‐POM: zinc polyoxometalate

## 1 Introduction

Diabetes mellitus (DM) is among the fastest‐growing chronic diseases worldwide in both mortality and healthcare expenditures. Currently, 589 million adults live with DM, and this number is projected to rise to 853 million by 2050 [1]. This growing prevalence imposes annual costs of hundreds of billions of US dollars on healthcare systems and the global economy [2]. Diabetic foot ulcer (DFU)—one of the most severe complications of DM—occurs at least once in the lifetime of roughly 25% of individuals with diabetes, and the five‐year mortality rate after major amputation exceeds that of many types of cancer [3]. Conventional approaches such as debridement, pressure off‐loading, antimicrobial treatments, and advanced dressings often fail because of chronic inflammation, impaired angiogenesis, and glycaemic dysfunction, making treatment both clinically and economically unsustainable, with recurrence rates exceeding 40% [4].

In tissue engineering, scaffolds are increasingly recognised not merely as mechanical or structural supports but also as active modulators of tissue regeneration through regulation of the local immune response. Acellular dermal matrix (ADM) scaffolds in full‐thickness skin wound models have been shown to activate the Lamtor1‐mediated acid‐sensing signalling pathway, thereby converting macrophages to the pro‐regenerative M2 phenotype and accelerating wound healing via matrix metalloproteinases (MMP‐3, MMP‐9) and growth factors (EGF, IGF, PDGF, TGF‐β, VEGFα) [5]. In bone tissue engineering, PLGA‐based scaffolds can perform osteo‐immunomodulatory functions. Yuan et al. [6] demonstrated that their PLGA hybrid bone scaffolds shifted the inflammatory microenvironment toward an anti‐inflammatory state, while Liu et al. [7] reported that porous PLGA/MBG (bioactive glass) scaffolds significantly enhanced bone regeneration and promoted a transition from the pro‐inflammatory M1 to the anti‐inflammatory M2 macrophage phenotype. Neutrophils on PCL scaffolds with different surface topographies were likewise found to modulate the osteogenic differentiation of mesenchymal stem cells (MSCs) [8]. Similarly, IL‐4‐loaded heparin hydrogels directed macrophages toward the M2 phenotype, creating a pro‐regenerative immune microenvironment and increasing osteogenesis in MC3T3‐E1 cells [9]. Liu et al. showed that collagen‐based scaffolds, including those derived from bovine skin, promoted healing in chronic wound models by reducing levels of inflammatory factors such as TNF‐α and IL‐1β and increasing anti‐inflammatory cytokines such as IL‐10 [10]. Recent designs of biomolecule‐containing hydrogels aim to control macrophage polarisation by tailoring physical parameters such as surface structure, stiffness, and porosity, thereby creating a more balanced inflammation–regeneration cycle in damaged tissue [11]. Moreover, a fully natural bioadhesive hydrogel enriched with protocatechuic aldehyde significantly accelerated angiogenesis and diabetic wound healing by regulating macrophage heterogeneity [12]. Collectively, these findings highlight scaffolds capable of regulating the immune response as a particularly promising strategy for treating DFUs, which are characterised by chronic inflammation and impaired healing.

Persistent impairment of healing in DFUs results from disruption of the normal sequential stages of wound repair and the establishment of a chronic inflammatory cycle [13]. In healthy tissue, pro‐inflammatory M1 macrophages dominate the early phase to clear pathogens and remove necrotic tissue, while a subsequent transition to the anti‐inflammatory M2 phenotype initiates tissue repair. In the diabetic microenvironment, however, this balance is lost: prolonged M1 activity and a cytokine storm with excessive production of TNF‐α, IL‐1β, and IL‐6 characterise the loss of M1/M2 polarisation [14, 15]. This persistent inflammatory stimulus delays granulation tissue maturation by suppressing fibroblast migration and keratinocyte proliferation [16].

Chronic hyperglycemia further exacerbates oxidative stress, leading to accumulation of advanced glycation end products (AGEs) and increased reactive oxygen species (ROS) generation. Mitochondrial dysfunction and reduced nitric oxide (NO) bioavailability weaken NO‐mediated signalling in endothelial cells, thereby suppressing vascular endothelial growth factor (VEGF) signalling and markedly limiting angiogenesis [17]. At the same time, diabetes‐related microangiopathy and tissue hypoxia impair the oxygen response via hypoxia‐inducible factor‐1α (HIF‐1α), while collagen cross‐linking and extracellular matrix (ECM) remodelling are disrupted [18, 19]. The combined effects of hyperglycaemia and hypoxia reduce the migratory capacity of dermal cells and reinforce the pro‐inflammatory phenotypes of neutrophils and macrophages, chronically locking wound healing in the inflammatory phase [20].

This pathological environment underscores the critical importance of immunomodulation. In DFU, it is not merely keratinocyte‐ or fibroblast‐based repair that fails; rather, the immune microenvironment itself governs tissue regeneration. Shifting macrophage polarisation from the pro‐inflammatory M1 to the anti‐inflammatory M2 phenotype, restoring ROS balance, and activating VEGF‐mediated vascular signalling not only reduce the inflammatory burden but also trigger angiogenesis, ECM remodelling, and functional tissue regeneration [15, 21]. Consequently, hydrogels that provide controlled IL‐4/IL‐10 release [22, 23] or nanofibers coated with nano‐enzymes to reduce ROS levels [24] should be viewed not as adjunctive therapies for DFU but as fundamental interventions that address the pathophysiological drivers of healing.

Recent advances in tissue engineering have enabled the development of active therapeutic platforms that extend beyond traditional dressings or surgical approaches for DFU management. Today, biomaterials are increasingly combined with cell‐ and gene‐based therapies to perform both regenerative and immunomodulatory functions [25].

Central to this transformation is the concept of “immune‐instructive biomaterials,” which can directly reprogram the immune response [26]. Next‐generation biomaterials promote cellular infiltration and reshape the inflammatory microenvironment by inducing macrophage transition from M1 to M2 phenotypes [27]. Biodegradable polymer scaffolds functionalised with MSC exosomes accelerate angiogenesis and the resolution of inflammation [28]. Collagen‐based scaffolds carrying small interfering RNA (siRNA) locally suppress pro‐inflammatory cytokines such as TNF‐α and IL‐1β, thereby regulating macrophage polarisation [29]. The incorporation of VEGF‐overexpressing fibroblasts into biodegradable scaffolds enhances local vascularisation while reducing oxidative stress, strengthening tissue regeneration [30]. Likewise, hypoxia‐responsive hydrogel systems stimulate angiogenesis and matrix remodelling under low oxygen conditions by activating HIF‐1α signalling [31]. Collectively, these innovations have transformed tissue‐engineering products from passive wound dressings into multifunctional therapeutic platforms that guide cell behaviour and the immune microenvironment, establishing a new paradigm in DFU treatment centred on immunomodulation.

Although the current literature highlights the advantages of immunomodulatory tissue‐engineering approaches for DFUs, the evidence base remains fragmented and heterogeneous, creating significant limitations. Most studies are confined to short‐term preclinical models [32, 33, 34, 35, 36, 37, 38, 39, 40, 41, 42, 43, 44, 45, 46, 47, 48, 49, 50, 51, 52, 53, 54, 55, 56, 57, 58, 59, 60, 61, 62, 63, 64]. High‐quality data on long‐term safety, biocompatibility, and functional tissue integration are scarce. Similarly, clear roadmaps for large‐scale manufacturing and cost‐effectiveness have yet to be established. Caravaggi et al. conducted a multicentre randomised controlled clinical trial showing that HYAFF 11–based autologous dermal and epidermal grafts significantly improved healing of dorsal DFUs compared with standard paraffin gauze dressings, whereas no additional benefit was observed for plantar ulcers due to the use of total off‐loading casts [65].

Regulation and good manufacturing practices (GMP) also represent critical barriers to clinical translation. Because bioengineering‐based products may fall under both medical device and Advanced Therapy Medicinal Product (ATMP) categories, they are subject to rigorous approval processes by regulatory agencies such as the U.S. Food and Drug Administration (FDA) [66] and the European Medicines Agency (EMA) [67]. Yet the current literature lacks a comprehensive framework offering practical guidance on how to meet these regulatory and GMP requirements.

There are studies in the literature that provide a more general, non‐systematic overview of biomedical interventions for inflammation modulation in diabetic wounds [68]. This systematic review focuses on methodologically synthesising DFU‐specific immunomodulatory tissue engineering strategies in accordance with PRISMA 2020 and presenting a mechanistic and translational framework through macrophage polarisation, oxidative stress/hypoxia, and cellular pathways.

This systematic review, by consolidating existing evidence within a single framework, addresses both fundamental immunological mechanisms and bioengineering applications from an interdisciplinary perspective. Specifically, the review explores the following research questions:

How do tissue‐engineering strategies modulate immune responses, particularly macrophage polarisation, in DFUs?

Which biomaterial or bioscaffold approaches best promote angiogenesis and reduce inflammation in DFUs?

How do cell‐ or gene‐based therapies influence immunomodulatory pathways in DFU healing?

Which key molecular pathways are most relevant for translating immunomodulatory findings in DFUs into clinical applications?

Answering these questions clarifies the current state of immunomodulatory tissue‐engineering applications in DFUs and provides a guiding framework for future innovative bioengineering strategies, translational research planning, and regulatory policy development. The aim of this systematic review is to systematically compile evidence from the past 5 years on bioengineering and regenerative medicine approaches targeting the DFU immune microenvironment and to evaluate these findings from a translational perspective to inform clinical applications.

## 2 Methods

### 2.1 Study Design

This systematic review was conducted in accordance with the *Preferred Reporting Items for Systematic Reviews and Meta‐Analyses (PRISMA) 2020* guidelines [69]. The objective was to identify and critically synthesise experimental and preclinical studies investigating *immunomodulatory tissue‐engineering strategies* for the management of *DFUs*.

### 2.2 Eligibility Criteria

Eligibility criteria were defined as a priori using the *PICOS framework* (Population, Intervention, Comparison, Outcomes, and Study design):

- *Population (P)*: Experimental models of DFUs or chronic diabetic full‐thickness wounds in humans or animals. Studies of other chronic wounds (e.g., venous or pressure ulcers) were excluded unless they reported a distinct diabetic subgroup.
- *Intervention (I)*: Any tissue‐engineering or biomaterial‐based strategy specifically aimed at *immunomodulation* of the wound microenvironment. Eligible interventions included hydrogels, scaffolds, nanocomposites, cell‐ or gene‐based therapies, and bioactive dressings designed to modulate immune or inflammatory responses.
- *Comparison (C)*: Conventional care, blank scaffolds or hydrogels, placebo, or other standard wound‐healing treatments used as controls. When no explicit comparator was reported, pre‐ and post‐intervention data from single‐arm studies were considered.
- *Outcomes (O)*: At least one immunomodulatory outcome—such as macrophage polarisation (M1/M2), cytokine or chemokine profiles, immune‐cell infiltration, or molecular indicators of immune‐microenvironment remodelling. Secondary outcomes included wound‐closure rate, collagen deposition, angiogenesis, and other markers of tissue regeneration.
- *Study design (S)*: Original experimental research including preclinical in vivo studies, in vitro mechanistic studies, and clinical trials (randomised or non‐randomised). Reviews, editorials, conference abstracts without primary data, and purely theoretical papers were excluded.

These criteria ensured that only studies directly addressing *immunomodulatory tissue‐engineering approaches* for DFU management were included.

### 2.3 Research Strategy

**FIGURE 1:**
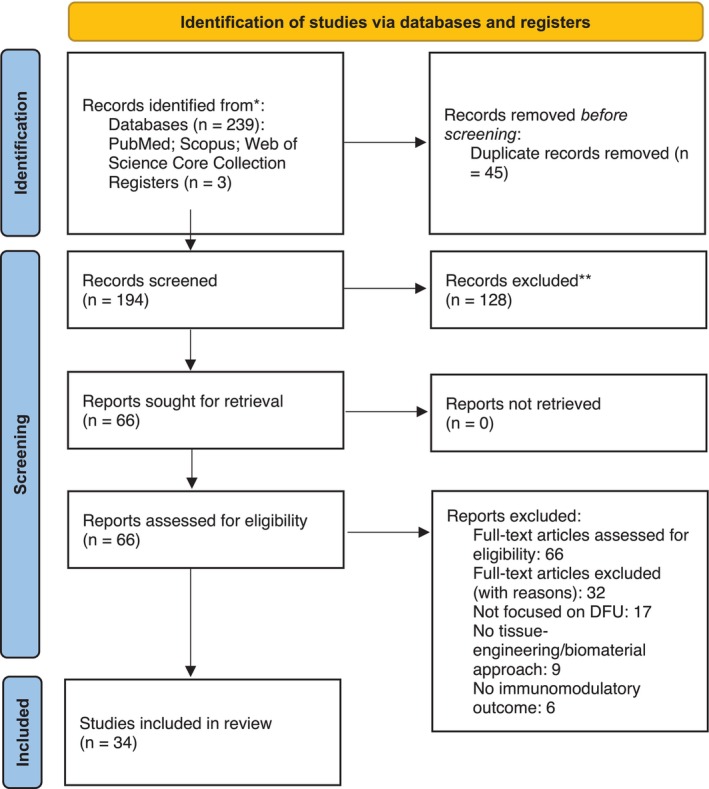
Flow diagram of the articles recovered and selected during the systematic review.

A comprehensive search of PubMed, Scopus, and Web of Science Core Collection was performed from database inception to 10 October 2025 (Figure 1). The search was restricted to articles published from 2020 to 2025, a period of rapid technological advances in immunomodulatory tissue engineering—particularly the emergence of immune‐instructive biomaterials and gene/cell‐based therapies with clinical potential. Older publications, while historically informative, often lacked standardised immunological endpoints and did not reflect the latest biomaterial design strategies. Limiting the timeframe ensured that the evidence synthesised is methodologically comparable and directly relevant to current translational and regulatory considerations, thereby strengthening the clinical roadmap offered by this review.

The search combined controlled vocabulary and free‐text terms representing three key concepts:

- DFU or chronic diabetic wound,
- tissue engineering or regenerative medicine, and
- immunomodulation of the wound microenvironment.

Search strings were tailored to each database; the full Boolean strategies and number of records retrieved are provided in Table S1. Reference lists of all included papers and relevant reviews were screened manually to capture additional studies. No language restrictions were applied.

### 2.4 Data Extraction

Two reviewers independently extracted data using a standardised, pre‐piloted form developed in accordance with PRISMA 2020 recommendations. For each eligible study, the following information was collected:

- *Bibliographic details*: first author, year of publication, and country of origin.
- *Study characteristics*: experimental model (in vivo, in vitro, or clinical), species or patient cohort, and sample size.
- *Intervention features*: type of tissue‐engineering or biomaterial approach (e.g., hydrogel, scaffold, cell or gene therapy), specific materials or bioactive compounds, and any adjunctive technologies (e.g., electrical stimulation, microfluidics).
- *Immunomodulatory outcomes*: markers of macrophage polarisation (M1/M2), cytokine or chemokine profiles, immune‐cell infiltration, and signalling‐pathway changes relevant to immune‐microenvironment modulation.
- *Regenerative outcomes*: wound‐closure rate and time to closure, angiogenesis markers (e.g., VEGF, CD31), collagen deposition, re‐epithelialisation, and other indicators of tissue regeneration.
- *Study conclusions and reported limitations*.

Data were extracted from full texts and, when necessary, from Supporting Information. Discrepancies were resolved through discussion or, when required, consultation with a third reviewer. When key quantitative or qualitative data were missing, corresponding authors were contacted. All extracted data were compiled in a *master evidence table* (Table 1) summarising the 34 included studies.

**TABLE 1:** Immunomodulatory tissue‐engineering strategies for diabetic foot ulcer management: Characteristics and key findings of the preclinical and clinical studies.

No.	First author (year)	Country/setting	Study type/model	Sample/cell type	Biomaterial/scaffold	Immunomodulatory strategy	Key outcomes	Main findings/conclusions	Limitations
12	Fu et al. (2023)	China–STZ‐induced diabetic Sprague–Dawley rat full‐thickness dorsal wound model with *S. aureus* infection	Development of all‐natural bioadhesive hydrogel (FGMA/FG/PA) consisting of fish gelatin methacrylate (FGMA), fish gelatin (FG), and protocatechuic aldehyde (PA); in vitro antioxidant/antibacterial/angiogenesis assays and in vivo diabetic wound healing	STZ‐induced diabetic SD rats (10‐mm full‐thickness dorsal wounds with *S. aureus* infection); RAW264.7 macrophages; HUVECs; L929 fibroblasts	FGMA/FG/PA hydrogel: physical FG/PA entanglement via Schiff base bonds + photo‐crosslinked FGMA network; bioadhesive, ROS‐scavenging, antibacterial	PA catechol and aldehyde groups scavenge ROS, provide intrinsic antibacterial activity and drive M1 → M2 macrophage polarisation (↓iNOS, ↑CD206), ↑ TGF‐β and VEGF, enhancing angiogenesis and collagen deposition	Strong bioadhesion to porcine skin: shear strength ~72 kPa; interfacial toughness ~173 J m^−2^ ROS scavenging: DPPH, OH, O_2_−radicals > 85% removed; intracellular ROS in RAW264.7 markedly ↓ *S. aureus* / *E. coli* survival < 20% at 8 h; antibacterial effect sustained > 48 h M1 → M2 shift: LPS‐stimulated RAW264.7: M1 ↓ from 57% to 22%, M2 ↑ to 40% HUVEC migration and tube formation ↑; VEGF secretion ↑ Diabetic rats: wound closure ~80% (Day 7) and ~94% (Day 21); ↑ collagen deposition, hair follicle regeneration, mature CD31^+^/α‐SMA^+^ vessels	All‐natural FGMA/FG/PA hydrogel provides safe, cost‐effective immunomodulation, promoting M2 macrophage polarisation, reducing inflammation, enhancing angiogenesis and accelerating infected diabetic wound healing	Preclinical animal study; active components' precise mechanisms and long‐term biosafety need further validation before clinical translation
32	Alizadeh et al. (2024)	Iran/STZ‐induced diabetic rat model; in vitro L929 fibroblasts and HUVECs	Emulsion electrospinning of core–shell micro/nanofibers; in vitro angiogenesis assays; in vivo diabetic wound healing	STZ‐diabetic rats (full‐thickness dorsal wounds); L929 fibroblasts; HUVECs	Emulsion‐electrospun PCL/SSA‐PVA core–shell scaffold loaded with 0.8% w/w copper oxide (CuO) nanoparticles	Sustained Cu^2+^ release up‐regulates VEGFA and bFGF expression in HUVECs and promotes CD31^+^ neovascularisation without eliciting immune rejection	CuO NP mean size 55–60 nm; zeta potential +37.8 mV Optimised 0.8% w/w CuO scaffold: tensile strength ≈1.6 MPa (dry), porosity ≈81%, sustained Cu^2+^ release ≈0.36 ppm over 21 days HUVEC tube formation and VEGFA/bFGF expression ↑1.3–3.3‐fold vs. control In diabetic rats: wound closure ≈89% at Day 21 vs. ≈60% in controls; ↑CD31 vessel density; enhanced re‐epithelialisation and hair‐follicle formation No adverse immune response in subcutaneous implantation; > 95% cell viability in L929 and HUVECs	The 0.8% w/w CuO core–shell scaffold provides controlled copper release that stimulates pro‐angiogenic signalling and markedly accelerates diabetic wound healing while maintaining excellent biocompatibility.	Preclinical animal study only; clinical translation and long‐term biosafety require further validation.
33	Bai et al. (2020)	China and Korea/STZ‐induced diabetic foot ulcer model in Sprague–Dawley rats; in vitro BM‐MSC culture	Preparation of injectable self‐healing hydrogel; in vitro BM‐MSC culture and growth factor secretion; in vivo diabetic foot ulcer healing in STZ‐induced diabetic rats	Male Sprague–Dawley rats (STZ‐induced diabetes; 5‐mm full‐thickness foot wounds, *n* = 45) Rat bone marrow mesenchymal stem cells (BM‐MSCs)	Self‐healing injectable hydrogel formed by in situ crosslinking of *N*‐carboxyethyl chitosan (*N*‐chitosan) and adipic acid dihydrazide (ADH) with hyaluronic acid‐aldehyde (HA‐ALD)	BM‐MSCs encapsulated in the hydrogel secrete TGF‐β1, VEGF and bFGF, inhibit M1 (CD86^+^) macrophage polarisation and enhance M2 (CD163^+^) polarisation, thereby regulating the chronic inflammatory microenvironment of diabetic wounds	Hydrogel gelation time ≈60 s; self‐healing and injectable properties Pore size 100–200 nm; swelling ratio ~75% in 120 min; complete biodegradation in 14 days BM‐MSCs in hydrogel secreted TGF‐β1 (34.7 ± 8.2 ng/L), VEGF (30.3 ± 2.8 ng/L), bFGF (3.58 ± 0.64 ng/L) at Day 9, all significantly higher than controls Wound area on Day 15: hydrogel + BM‐MSCs group ≈14% residual vs. ≈42% in controls Increased Ki67^+^ cell proliferation, CD31^+^ neovascularisation, collagen deposition and granulation tissue formation	Injectable N‐chitosan/HA‐ALD self‐healing hydrogel encapsulating BM‐MSCs modulates chronic inflammation, promotes secretion of angiogenic and pro‐healing growth factors, accelerates granulation tissue formation, neovascularisation and collagen deposition, and significantly enhances healing of STZ‐induced diabetic foot ulcers	Preclinical animal study; long‐term safety and clinical efficacy remain to be demonstrated before translation to human diabetic foot ulcer therapy
34	Bakadia et al. (2024)	China–in vivo diabetic wound models (rat and mouse)	– Development and characterisation of teicoplanin‐decorated reduced graphene oxide (rGO) incorporated silk protein (SP) hybrid hydrogel crosslinked with genipin. – In vitro antibacterial/antibiofilm, cytocompatibility, and cell migration assays. – In vivo: STZ‐induced diabetic rats (non‐infected wounds) and *S. aureus* ‐infected diabetic mice (diabetic foot osteomyelitis model)	STZ‐induced diabetic Sprague–Dawley rats (full‐thickness dorsal wounds). *S. aureus* ‐infected BALB/c diabetic mice (infected wound and tibial osteomyelitis model). NIH‐3T3 fibroblasts, HUVEC endothelial cells, MC3T3 osteoblast‐like cells	Teicoplanin‐decorated reduced graphene oxide (rGO) incorporated silk protein (sericin + fibroin) hybrid hydrogel, chemically and physically crosslinked with genipin (GNP)	Hybrid hydrogel down‐regulates pro‐inflammatory cytokines (TNF‐α, IL‐1β, IL‐6), up‐regulates anti‐inflammatory cytokines (IL‐10, IL‐4), reduces MMP‐9 expression, and promotes M2 macrophage polarisation and angiogenesis; teicoplanin provides potent antibacterial and antibiofilm activity with high bone‐penetration to prevent diabetic foot osteomyelitis	Porosity 70%–80%, average pore size ≈190 ± 81 nm; high self‐healing and shear‐thinning properties. Antibiofilm activity: biofilm eradication on hydroxyapatite disks with penetrability index 86%–68% for *S. aureus* , 71%–52% for MRSA, 62%–42% for *E. coli* , 59%–29% for *P. aeruginosa* . Excellent hemocompatibility (< 0.5% hemolysis) and > 95% NIH‐3T3 and HUVEC cell viability. Fibroblast migration: wound closure 97% ± 2.8% in vitro at 48 h. In vivo: ~100% wound closure by Day 14 in both diabetic rat and infected mouse models; marked reduction of pus and *S. aureus* infection; improved tibial bone regeneration in osteomyelitis model	The teicoplanin‐decorated rGO–silk protein hybrid hydrogel exhibits strong antibacterial and antibiofilm properties, immunomodulatory effects (M2 polarisation, cytokine balance), and pro‐angiogenic activity, which together accelerate healing of infected diabetic wounds and prevent diabetic foot osteomyelitis, while maintaining excellent biocompatibility	Preclinical animal study; long‐term safety, pharmacokinetics of teicoplanin‐decorated rGO, and clinical efficacy require further validation before translation to human diabetic foot ulcer therapy
35	Chen et al. (2023)	China–preclinical diabetic mouse wound model and pilot clinical trial in patients with diabetic foot ulcers (DFUs)	Development of an injectable nano‐platelet vesicle–based extracellular matrix hydrogel (NPV‐ECM). In vitro macrophage metabolic assays and HUVEC angiogenesis assays. In vivo STZ‐induced diabetic mouse full‐thickness wound model. Phase‐I investigator‐initiated clinical trial in six DFU patients (ChiCTR2100046769)	LPS‐activated bone marrow–derived macrophages (BMDMs). Human umbilical vein endothelial cells (HUVECs). STZ‐induced diabetic mice (6‐mm dorsal wounds). Six DFU patients (63.2 ± 8.8 years.; wounds non‐healing ≥ 8 weeks)	Autologous nano‐platelet vesicle–extracellular matrix (NPV‐ECM) hydrogel, prepared by extruding donor platelets into 34 nm NPVs and mixing with plasma fibrinogen; in situ thrombin‐triggered gelation	Selective nutrient restriction (“hyperinflammatory starvation therapy”): hydrogel limits diffusion of pro‐inflammatory metabolites (e.g., glucose, succinate) while permitting lipid transport. Reprograms macrophage metabolism: ↓ glycolysis and IL‐6 signalling; ↑ TCA cycle, oxidative phosphorylation and fatty acid oxidation. Promotes M2 macrophage polarisation and angiogenesis	NPVs size ≈34 nm; zeta potential −24.4 mV; > 95% NPVs released from hydrogel within 72 h. In vitro: glucose consumption and lactate production ↓; ATP:ADP ratio ↓; pro‐inflammatory gene expression (Il6, Nos2, Tnfa) ↓; anti‐inflammatory genes (Arg1, Il10, Cd206) ↑. HUVEC migration ↑~80% and tube formation ↑ vs. control. Diabetic mice: wound area reduced to ~2.4‐fold smaller than untreated by Day 12; ↑ CD31^+^ neovascularisation and NF200^+^ nerve fibres; faster re‐epithelialisation and collagen deposition. Clinical pilot (*n* = 6 DFU patients): relative wound area ↓ from 100% to 46.4% ±29.3% at 4 weeks (*p* = 0.0094); mean closure time 63 ± 10 days; no serious adverse events	NPV‐ECM provides a biomaterial‐based “hyperinflammatory starvation therapy,” selectively restricting pro‐inflammatory nutrient uptake, rewiring macrophage metabolism toward a reparative phenotype, enhancing angiogenesis and nerve regeneration, and accelerating healing of diabetic wounds in both mice and a first‐in‐human DFU pilot trial	Pilot clinical trial with only six patients and short follow‐up; long‐term safety, large‐scale efficacy, and regulatory validation remain necessary before broad clinical application
36	Chen et al. (2024)	China–STZ‐induced diabetic rat full‐thickness wound model	– Design and evaluation of dynamic hyaluronic acid (HA) hydrogels cross‐linked via boronate and Zn2+ coordination chemistry. – In vitro antioxidation, anti‐inflammatory and angiogenesis assays (L929 fibroblasts, HaCaT keratinocytes, RAW264.7 macrophages, HUVECs). – In vivo full‐thickness STZ‐diabetic rat wound healing	STZ‐induced diabetic Sprague–Dawley rats (10‐mm dorsal wounds). L929 fibroblasts, HaCaT keratinocytes, RAW264.7 macrophages, HUVECs	Dynamic HA hydrogel (HTZS) prepared by boronate and Zn^2+^ coordination chemistry, incorporating HA‐H_2_S donor and tannic acid (TA) to enable ROS/glucose/pH‐responsive degradation and controllable H_2_S release	“Troika” mechanism: ROS elimination + H_2_S release + Zn2+ regulation. Promotes macrophage M1 → M2 repolarisation, down‐regulates pro‐inflammatory genes (TNF‐α, IL‐1β, IL‐6, iNOS) and up‐regulates anti‐inflammatory genes (IL‐4, IL‐10, Arg1, CD206). Activates PI3K–Akt signalling to enhance angiogenesis and regulate glucose/tyrosine metabolism	Fast gelation (~5 s); self‐healing and injectable; mean pore size 6.6 ± 2.3 μm; swelling ratio ~70%–80% in 2 h. Responsive degradation: t½ = 20 h (11.1 mM glucose), 5 h (250 μM H_2_O_2_), 1.5 h (pH 5). Continuous H_2_S release ≈40 μM in 48 h; Zn2+ release 50%–60% in 12 h. In vitro: ROS level ↓ to 7.8% (vs. 93.7% positive control); CD86+ M1 ↓ to 11.3%; CD206+ M2 ↑ to 18%; HUVEC tube formation and HaCaT migration ↑ (scratch closure 78.9%). In vivo: wound closure 98.9% by Day 12 (vs. 79.6% Tegaderm on Day 14); full re‐epithelialisation; normalised epidermis/dermis; dense hair follicle regeneration. Upregulation of angiogenesis‐related genes (CD31, VEGF, eNOS, HIF‐1α) and collagen‐I/III ratio ↑ to 3.5	Dynamic HA hydrogel (HTZS) provides a multi‐pronged therapy—ROS scavenging, sustained H_2_S release and Zn^2+^‐mediated metabolic/angiogenic regulation—leading to M1 → M2 macrophage repolarisation, enhanced neovascularisation, high collagen‐I deposition and hair follicle regeneration, thereby achieving complete structural and functional healing of diabetic wounds within 12–14 days	Preclinical animal study; long‐term safety, large‐scale efficacy and regulatory validation are still required before clinical translation
37	Chu et al. (2025)	China–STZ‐induced diabetic rat foot infection wound model	Development of self‐healing polypyrrole (Ppy)–based hybrid hydrogel composed of hyaluronic acid (HA), poly(vinyl alcohol) (PVA), and polyethylene glycol (PEG). In vitro antibacterial and macrophage cytokine assays (RAW264.7 cells). In vivo S. aureus–infected diabetic foot wound model in STZ‐induced diabetic rats under near‐infrared (NIR, 808 nm) photothermal stimulation	STZ‐induced diabetic male Sprague–Dawley rats (6‐mm full‐thickness foot wounds, infected with 1 × 108 CFU/mL S. aureus). RAW264.7 murine macrophages for cytokine analysis	Self‐healing HA/PVA/PEG hybrid hydrogel containing polypyrrole (Ppy) nanoparticles (TPHB formulation, 2:1:1:2 Ppy:HA:PEG:PVA ratio), fabricated by repeated freeze–thawing	Two‐stage immune orchestration triggered by NIR photothermal therapy: – NIR‐induced heat (~67°C in air; ~58°C in water) activates DAMPs and complement receptor system, enhances macrophage phagosome activity, and promotes M1 (CCR7^+^) macrophage activation with high TNF‐α secretion to accelerate bacterial clearance. – Following infection control, macrophage phenotype shifts to M2 (CD206^+^) with increased TGF‐β expression, promoting anti‐inflammatory signalling, angiogenesis (↑VEGF) and tissue remodelling	Photothermal antibacterial effect: complete eradication of *S. aureus* biofilm under NIR (0.5 W/cm^2^) within 10 min; C3 complement gene expression significantly ↑. RAW264.7 cells: TNF‐α secretion peaks at 0.5 W/cm^2^ NIR; TGF‐β slightly ↑ after infection clearance. In vivo wound healing: NIR‐treated TPHB hydrogel achieved highest healing rate; wound closure significantly ↑ vs. control at days 3, 7 and 14; marked reduction in bacterial load. Histology: VEGF overexpression, dense collagen deposition, increased epidermal thickness, up‐regulated genes for keratinisation and skin barrier formation.	NIR‐triggered Ppy hybrid hydrogel provides rapid photothermal sterilisation and complement‐mediated macrophage activation (M1), followed by a natural switch to anti‐inflammatory M2 polarisation, resulting in enhanced VEGF expression, collagen deposition, and complete healing of *S. aureus* –infected diabetic wounds	Preclinical animal study; long‐term biosafety, large‐scale validation, and clinical translation remain to be established
38	Geng et al. (2025)	China–STZ‐induced diabetic rat full‐thickness wound model	Development and characterisation of a self‐healing puerarin–borax–polyvinyl alcohol (PVA) hydrogel (BP hydrogel). In vitro biocompatibility (L929 fibroblasts), hemolysis and antibacterial assays. In vivo STZ‐induced diabetic rat chronic wound model; transcriptomic (RNA‐seq) and multiple immunohistochemistry (mIHC) analyses	Male STZ‐induced diabetic Sprague–Dawley rats (8‐mm dorsal full‐thickness wounds). L929 fibroblasts for cytocompatibility testing	Self‐healing PVA–borax–puerarin (BP) hydrogel formed by borate ester bonds; optimised composition: 5 wt% PVA, 1 wt% borax, 1 wt% puerarin	Synergistic immunomodulation and tissue‐regenerative remodelling: – Early phase: ↓ iNOS^+^ M1 macrophages, ↑ CD206^+^ M2 macrophages (*p* < 0.0001). – Enhanced angiogenesis: ↑ CD31^+^ and VEGFA^+^ vessel density (*p* < 0.001). – Collagen remodelling: ↑ Type I and III collagen deposition (*p* < 0.05). RNA‐seq: downregulation of pro‐inflammatory pathways (e.g., JAK–STAT, Tnf), upregulation of genes related to keratinocyte differentiation and epidermis development	Wide linear viscoelastic range (398%), compressive strength 4310 Pa; self‐healing and high ductility (stretchable to ~1 m). Hemolysis rate < 5% (ASTM F756‐17 standard); > 95% L929 cell viability. Antibacterial activity against Staphylococcus aureus with concentration‐dependent killing. Diabetic rats: wound healing rate significantly ↑ vs. model from Day 3 onward; by Day 10, nearly complete epithelialisation, dense collagen, abundant neovasculature and hair follicle regeneration	The self‐healing puerarin–borax–PVA hydrogel improves puerarin solubility and provides mechanical robustness, excellent biocompatibility, and antimicrobial activity. Through macrophage M1 → M2 repolarisation, pro‐angiogenic VEGFA/CD31 induction, and collagen I/III remodelling, it markedly accelerates chronic diabetic wound healing	Preclinical animal study only; long‐term biosafety of borax‐containing formulation and industrial‐scale production feasibility require further evaluation before clinical translation.
39	Guo et al. (2024)	China–STZ‐induced type I diabetic rat full‐thickness wound model	Microfluidic fabrication of ***Bletilla striata*** *polysaccharide (BSP) methacrylate (BSPMA)* hydrogel microspheres loaded with *20(S)‐protopanaxadiol (PPD) liposomes* (PPD‐Lipo@HMs); in vitro cell migration/angiogenesis and macrophage polarisation assays; in vivo diabetic wound healing	STZ‐induced diabetic Sprague–Dawley rats (8 mm dorsal full‐thickness wounds); L929 fibroblasts, HUVECs, RAW264.7 macrophages	Microfluidic‐engineered *PPD‐Lipo@BSPMA hydrogel microspheres*, photocrosslinked; average microsphere diameter ≈332 μm; PPD encapsulation efficiency 76.99% ± 10.01%	BSP targets macrophage mannose receptors, promoting *M1* → *M2* *macrophage polarisation*; PPD activates *PI3K/Akt/mTOR and Raf/MEK/ERK* pathways, stimulating VEGF secretion and angiogenesis; combined effect reduces inflammation, enhances re‐epithelialisation and granulation tissue formation	Swelling ratio: HMs 1843%, PPD‐Lipo@HMs 1658% in 30 min; sustained structure over time Encapsulation efficiency: PPD‐Lipo 87.38% ± 10.51%, PPD‐Lipo@HMs 76.99% ± 10.01% Hemolysis < 5%; L929 and HUVEC viability > 90% L929 and HUVEC migration and tube formation significantly ↑ vs. control RAW264.7 M2 macrophages: 7.62% (vs. 0.15% control) Diabetic rats: wound healing 94.22% Day 14 vs. 84.76% control; collagen deposition 56.3% ± 3.6% (vs. 37.7% ± 3.5% control); CD31+ vessel density and α‐SMA+ angiogenesis markers markedly ↑	Microfluidic‐engineered PPD‐Lipo@HMs provide *dual herbal–nanoliposome therapy*, combining BSP‐mediated immune modulation and PPD‐driven pro‐angiogenic signalling, leading to accelerated collagen deposition, robust angiogenesis and nearly complete wound closure of diabetic skin injuries	Preclinical animal study; long‐term biosafety of PPD‐Lipo@HMs and large‐scale clinical validation are required before translation
40	Hao et al. (2023)	Taiwan–STZ‐induced diabetic rat full‐thickness dorsal wound model	Development of Gelatin–Alginate (GelAlg) hydrogel loaded with reduced graphene oxide (rGO) and human platelet‐derived extracellular vesicles (pEVs) (GelAlg@rGO‐pEV); in vitro cytocompatibility, ROS‐scavenging, macrophage polarisation, and cell migration assays; in vivo diabetic wound healing with near‐infrared (NIR) photothermal activation	STZ‐induced diabetic Wistar rats (8 mm dorsal full‐thickness wounds); L929 fibroblasts; RAW264.7 macrophages	Photothermally responsive GelAlg@rGO‐pEV hydrogel: Gel 10% + Alg 7.5% + rGO 200 μg mL^−1^ + pEVs 2 mg mL^−1^; porous scaffold (~61% porosity) with enhanced compressive modulus (106 kPa)	pEVs provide platelet‐derived trophic factors (VEGF, PDGF, TGF‐β) that promote M1 → M2 macrophage polarisation and reduce pro‐inflammatory cytokines; rGO enables NIR‐triggered mild photothermal hyperthermia which induces heat shock protein (HSP) expression, augments angiogenesis and follicle regeneration, and enhances ROS scavenging	Compressive modulus ↑ from 78 kPa (GelAlg) to 106 kPa (GelAlg@rGO) > 80% L929 viability; no organ toxicity Intracellular ROS in L929 and RAW264.7 ↓ significantly vs. LPS control M2/M1 ratio restored to control levels after pEVs treatment NIR (808 nm, 2 W cm−2, 5 min) induced HSP expression and VEGF/CD31 angiogenesis markers Complete wound closure by Day 14 vs. ~65% remaining wound in PBS control	NIR‐activated GelAlg@rGO‐pEV hydrogel combines ROS scavenging, immunomodulation (M2 polarisation), and HSP‐driven angiogenesis, producing complete healing of chronic diabetic wounds with no systemic toxicity	Preclinical animal study; clinical‐scale production of clinical‐grade pEVs and long‐term safety require further evaluation before human application
41	Hauck et al. (2021)	Germany–db/db diabetic mouse chronic wound model	Development of hyaluronan–acrylate/collagen (HA‐AC/coll) hydrogels releasing high‐sulfated hyaluronan (sHA); in vitro macrophage activation assays, skin ex vivo cultures, imiquimod (IMQ)‐induced acute skin inflammation in mice, and in vivo chronic wound healing in diabetic db/db mice	Murine peritoneal tissue‐resident macrophages and bone marrow–derived macrophages; C57BL/6 skin biopsies; BALB/c mice (IMQ skin inflammation); diabetic db/db mice (6‐mm full‐thickness dorsal wounds)	Photo‐crosslinked HA‐AC/collagen hydrogel incorporating high‐sulfated hyaluronan (sHA) for sustained release (~100 μg over 8 days)	sHA binds CD44, CD36, LOX‐1 on macrophages → inhibits TLR/NFκB/STAT1 signalling, ↓ pro‐inflammatory cytokines (IL‐1β, TNF, IL‐6, CCL2), ↓ NLRP3 inflammasome activation; shifts macrophages toward anti‐inflammatory phenotype (↑ IL‐10, IL‐1RA, RELMa)	In vitro: sHA ↓ IL‐1β, TNF, IL‐6, CXCL1, CCL2 gene expression; ↓ IL‐1β release even under palmitic acid–induced inflammasome activation Ex vivo skin: ↓ keratinocyte S100A8/A9 expression IMQ‐induced skin inflammation: ↓ epidermal thickening, ↓ Ki67+ proliferation, ↓ CD11b+ infiltration; ↑ IL‐10 and IL‐1RA in CD11b+ cells Diabetic db/db wounds: ↓ IL‐1β, S100A9, NLRP3, ↑ RELMa, IL‐10, HO‐1; enhanced M2 macrophage activation; ↑ CD31+ neovascularisation; accelerated granulation and re‐epithelialisation; significant wound closure by Day 10	sHA‐releasing HA‐AC/coll hydrogels provide sustained local immunomodulation, dampen chronic inflammation, promote M2 macrophage polarisation and vascularisation, and significantly accelerate healing of diabetic wounds	Preclinical animal study; long‐term biosafety, human translational studies and large‐scale manufacturing require further evaluation
42	He et al. (2024)	China–STZ‐induced diabetic mouse full‐thickness wound model	Electrospun polycaprolactone/gelatin (P/G) nanofiber membrane covalently grafted with chitosan–4‐Octyl itaconate (CS‐OI); in vitro anti‐inflammatory/antioxidant assays; in vivo diabetic wound healing	STZ‐induced diabetic male ICR mice (8 mm dorsal full‐thickness wounds); RAW264.7 macrophages; HaCaT keratinocytes	P/G‐CS‐OI nanofiber membrane with sustained OI release (~75% in first 2 weeks, total 28 days), fibre diameter ≈371 ± 91 nm; UTS 20 ± 2 MPa	OI alkylates KEAP1, activates NRF2/ARE antioxidant pathway, ↓ ROS and pro‐inflammatory genes (TNF‐α, IL‐1β, IL‐6), ↑ IL‐10, IL‐4; promotes M1 → M2 macrophage polarisation (CD86↓, CD206↑), enhances VEGF secretion and keratinocyte migration	Hemolysis < 2%; > 90% RAW264.7 and HaCaT viability ROS level ↓ significantly vs. LPS control; SOD ↑; NO ↓ NRF2 and downstream HO‐1, NQO1, GCLC, GCLM ↑ M2 macrophages ↑ to 20.84% vs. 7.26% M1 (flow cytometry) Day‐14 wound closure ~100% vs. delayed healing in controls; dense collagen, mature CD31+/α‐SMA+ vessels, complete re‐epithelialisation with strong K10+/K14+ layers	P/G‐CS‐OI membrane provides sustained antioxidative and anti‐inflammatory activity, reprograms macrophage metabolism toward M2 phenotype and markedly accelerates diabetic wound healing with mature angiogenesis and epithelial regeneration	Preclinical animal study; long‐term biosafety and clinical translation need further validation
43	Kim et al. (2024)	South Korea–induced diabetic C57BL/6 mouse full‐thickness dorsal wound model	Fabrication of Zn/AgCl redox‐patterned polyurethane (PU) membrane layered with electrospun PVA/gelatin (2.5%) nanofibers (“M‐sheet”) that spontaneously generates microcurrents; in vitro antibacterial, cell migration and macrophage polarisation assays; in vivo diabetic wound healing	STZ‐induced diabetic male C57BL/6 mice (6‐mm dorsal full‐thickness wounds); HaCaT keratinocytes; human dermal fibroblasts (HDFs); RAW264.7 macrophages	M‐sheet: screen‐printed Zn/AgCl electrodes on PU film overlaid with PVA/gelatin nanofibers; generates self‐sustained electric field (0.45 V mm^−1^ in 5 min; > 0.2 V mm^−1^ for 3 days); sustained Zn and Ag ion release (Zn 154 → 655 μg L^−1^; Ag 291 → 385 μg L^−1^ over 5 days)	Electric‐field–mediated stimulation induces M1 → M2 macrophage polarisation (↓iNOS, ↑CD206), ↓ pro‐inflammatory cytokines, ↑ VEGF/TGF‐β; promotes keratinocyte/fibroblast migration and angiogenesis	Strong antibacterial activity vs. E. coli and S. aureus; lowest CFUs among controls HaCaT and HDF scratch closure after 24 h: remaining gap 27.6% and 10.1% RAW264.7: M2 (CD206+) cells significantly ↑; iNOS expression ↓ Diabetic mice: wound closure 96.4% ± 0.97% by Day 24 vs. 79.5% Bare, 72.6% control H&E/MTC: narrow panniculus gap, thick granulation, dense collagen, high skin appendage regeneration Immunofluorescence: ↑ Ki67, cytokeratin‐10, α‐SMA, CD31 indicating proliferation, re‐epithelialisation, and neovascularisation	Battery‐free M‐sheet dressing provides continuous microcurrent‐driven antibacterial action and immunomodulation, accelerating re‐epithelialisation, collagen synthesis and angiogenesis, leading to near‐complete healing of diabetic wounds	Preclinical animal study; long‐term biosafety, large‐scale manufacturing and human clinical validation still required
44	Lan et al. (2024)	China–STZ‐induced diabetic rat dorsal full‐thickness wound model with MRSA and multidrug‐resistant *P. aeruginosa* (MRPA) infection	Development of HAQ hydrogel: hyaluronic acid methacrylate (HAMA) modified with 3‐(acrylamido)phenylboronic acid (AAPBA) and quaternary ammonium chitosan (QCS); in vitro antibacterial, biofilm, hemocompatibility, and cell cytotoxicity assays; in vivo haemostasis and infected diabetic wound healing	STZ‐induced diabetic male Sprague–Dawley rats (8‐mm dorsal wounds infected with MRPA 1 × 10^7^ CFU/mL); rat skin fibroblast‐like cells (RS1), L929 fibroblasts, RAW264.7 macrophages	Injectable HAQ hydrogel photocrosslinked from HAMA/AAPBA/QCS; average pore size ~30 μm; tensile strength 66.99 kPa; swelling ratio 1015%; water retention high; hemostatic activity in liver and tail bleeding models	Phenylboronic acid groups rapidly capture bacteria via reversible borate–diol bonds while QCS provides bactericidal activity by disrupting bacterial membranes; together reduce inflammation and enable early transition from inflammatory to proliferative healing phase	MRSA survival reduced by 75% (HAQ1) and 100% (HAQ2); MRPA survival 75% (HAQ1) and 99.96% (HAQ2) in vitro Biofilm biomass ↓ to 18.72% (MRSA) and 37.95% (MRPA) vs. control Hemolysis < 5%, > 80% RS1, L929, RAW264.7 viability Tail bleeding blood loss ↓ to 16.33 mg vs. 59 mg gauze control Diabetic infected wounds: wound area only 15.6% remaining by Day 14 vs. > 30% in controls; TNF‐α expression markedly ↓, CD31+ neovascularisation ↑	HAQ hydrogel exhibits dual‐function rapid bacterial capture and killing, potent anti‐biofilm and hemostatic effects, promotes angiogenesis and significantly accelerates healing of drug‐resistant bacteria‐infected diabetic wounds	Preclinical animal study; long‐term biosafety and clinical translation require further validation
45	Li et al. (2025)	China–STZ‐induced type I diabetic rat full‐thickness dorsal wound model	Development of portable electrospinning dressing (PED4) loaded with kaempferol; in vitro cytocompatibility and in vivo diabetic wound healing evaluation	STZ‐induced diabetic male Sprague–Dawley rats (8 mm dorsal full‐thickness wounds); NIH/3 T3 fibroblasts	PED4 nanofiber dressing: 5% PVB + 1% kaempferol +15% Pluronic F‐127 electrospun in situ using portable device; fibre diameter ~2.28 μm; WVTR 4.88 kg m^−2^·24 h^−1^	Kaempferol downregulates MMP9 expression via NF‐κB inhibition; promotes M1 → M2 macrophage polarisation (CD86↓, CD206↑); reduces pro‐inflammatory cytokines (TNF‐α, IL‐1β, IL‐6) and enhances anti‐inflammatory IL‐10; stimulates CD31^+^/VEGF^+^ neovascularisation	> 90% NIH/3 T3 viability; excellent biocompatibility WVTR suitable for moist wound healing (4.88 kg·m^−2^·24 h^−1^) Day‐15 wound closure 95.9% vs. markedly less in control/gauze Collagen deposition markedly ↑ (dense, organised fibres) MMP9 fluorescence intensity significantly ↓ CD206+ M2 macrophages ↑; pro‐inflammatory cytokines TNF‐α, IL‐1β, IL‐6 ↓; IL‐10 ↑ CD31+ and VEGF+ neovascularisation significantly ↑	Portable kaempferol‐loaded PED4 dressing provides personalised in situ electrospinning therapy, simultaneously reducing MMP9 activity, reprogramming macrophages toward an anti‐inflammatory phenotype, promoting angiogenesis and collagen deposition, and achieving rapid diabetic wound closure	Preclinical animal study; clinical translation and long‐term safety of portable electrospinning dressings require further validation
46	Liu et al. (2025)	China–STZ‐induced diabetic foot ulcer (DFU) C57BL/6 mouse full‐thickness wound model	Development of trehalose‐modified alginate–polyacrylamide hybrid hydrogel (MGTSe) containing zero‐valent selenium nanoparticles (Se NPs); in vitro antioxidant, antibacterial, macrophage polarisation and selenoprotein synthesis assays; in vivo DFU healing and microbiota‐immune homeostasis evaluation	STZ‐induced diabetic C57BL/6 mice (8‐mm full‐thickness dorsal wounds); RAW264.7 macrophages; HUVECs	MGTSe hydrogel: trehalose‐modified alginate–polyacrylamide interpenetrating network with in situ synthesised Se NPs (~80 nm); Se release ≈66 μg/24 h; porosity ~62.5%; strong skin adhesion (peel strength 2.94 N); tensile strength 39.1 kPa	Se NPs enable in situ selenoprotein synthesis (GPX1/2/4, GPXW) via SeCys2 pathway; selenoproteins inhibit NF‐κB signalling (↓p65, p‐IκBα, p‐IKKα/β), ↓pro‐inflammatory cytokines (TNF‐α, IL‐1β, IL‐6); promote M1 → M2 macrophage polarisation (CD86↓, CD206↑); trehalose reduces bacterial adhesion and helps maintain microbiota balance (↑Lactobacillus, ↓ *E. coli* and *S. aureus* )	> 90% HUVEC and RAW264.7 viability; strong ROS scavenging (intracellular ROS ↓; SOD ↑) M2 macrophages ↑; pro‐inflammatory cytokines ↓ to near‐normal levels • Selenoproteins GPX1/2/4, GPXW markedly ↑ In vivo: wound area ↓ to 7.3% by Day 10 vs. 47.2% diabetic control; epidermal thickness normalised; dense collagen, mature CD31+/VEGF+ neovasculature; MMP‐9 expression ↓; IL‐1β and IL‐6 expression ~0.5× of control; skin microbiota: ↑probiotic Lactobacillus, ↓pathogenic *E. coli* and *S. aureus*	MGTSe hydrogel provides biochemical strategy‐based immune–microbiota co‐regulation via in situ selenoprotein synthesis, effectively restoring microbial–immune balance and accelerating DFU healing with robust angiogenesis and collagen deposition	Preclinical animal study; long‐term biosafety of Se NPs and clinical translation require further validation
47	Mai et al. (2025)	China–db/db diabetic mouse full‐thickness dorsal wound model	Catalyst‐modulated dynamic lysozyme–PEG (LZM‐HZ) hydrogel with tunable viscoelasticity via reversible acylhydrazone crosslinking; in vitro macrophage migration/polarisation and angiogenesis assays; in vivo diabetic wound healing, transcriptomic and mechanistic analyses	db/db type II diabetic mice (8‐mm full‐thickness dorsal wounds); RAW264.7 macrophages; HUVECs	LZM‐HZ dynamic hydrogel crosslinked between aldehyde‐modified lysozyme (LZM‐CHO) and 4‐arm PEG‐hydrazide, with 4‐amino‐DL‐phenylalanine (4a‐Phe) catalyst to modulate bond exchange kinetics; tunable half‐relaxation time (τ1/2) 50 → 15 min at constant storage modulus (~850 Pa)	Enhanced network dynamics activate JAK/STAT signalling, promoting macrophage M1 → M2 polarisation (↓CD86, IL‐1β; ↑CD204, VEGF), ↑ macrophage migration and spreading; macrophage‐conditioned medium increases HUVEC tube formation; in vivo upregulates Arg1, TGFβ1, VEGFA, and angiogenesis‐related genes while suppressing pro‐inflammatory genes	Gelation time shortened from 16 h → 4 min with higher catalyst G′ ~850 Pa constant; G″ ↑17 → 120 Pa τ1/2 50 → 15 min as catalyst ↑ > 90% RAW264.7 viability; strong self‐healing and adhesion (~10 kPa) In vivo: wounds nearly fully epithelialised by Day 11; dense collagen and mature vessels comparable to PDGF‐BB positive control; increased CD206+ M2 macrophages and CD31+ neovascularisation; Western blot: ↑p‐STAT3, p‐STAT6, Arg1	Catalyst‐regulated dynamic hydrogel decouples viscoelasticity from stiffness and intrinsically drives JAK/STAT‐mediated immune modulation, creating a pro‐regenerative microenvironment that accelerates diabetic wound closure with high‐quality collagen deposition and vascularisation	Preclinical animal study; long‐term biosafety of 4a‐Phe catalyst and large‐scale clinical translation require further validation
48	Ning et al. (2022)	China– *S. aureus* –infected STZ‐induced diabetic SD rat chronic wound model	Design of dual‐layer synergistically detachable microneedle (DDMNS) dressing composed of chitosan hydrogel dressing (CSHD) and detachable microneedle patch with Panax notoginseng saponins (PNS)–loaded chitosan (CS) tip and magnesium (Mg) particle–loaded polyvinylpyrrolidone (PVP) backing; in vitro antibacterial/biocompatibility and in vivo programmed wound healing evaluation	*S. aureus* –infected chronic wounds in STZ‐induced diabetic Sprague–Dawley rats; NIH‐3 T3 fibroblasts	DDMNS: upper CSHD + lower detachable PNS‐CS/Mg‐PVP microneedle array; Mg‐PVP backing dissolves rapidly in moist CSHD, enabling burst drug release and tip implantation for sustained PNS release	Mg particles react with inflammatory microenvironment to release H_2_ and Mg^2+^, synergistically accelerating microneedle detachment and inhibiting inflammation; Mg^2+^ promotes M1 → M2 macrophage polarisation (↑CD206/IL‐1β ratio) and reduces IL‐6 and TNF‐α; PNS provides prolonged pro‐angiogenic activity	Detachment ratio 87.5% (Mg+/CSHD+) within 5 min vs. 15.2% control Burst release of model drugs (RhB, BSA) 89%–90% within 15 min from Mg‐PVP layer; PNS from CS tip sustained 7 days Antibacterial effect: 86.1% E. coli and 95.7% S. aureus killing Day‐10 wound area 6.34% vs. 34.43% control; granulation thickness 1271.7 μm vs. 437.4 μm control CD31+ neovascularisation 8.51% vs. 2.27% control; IL‐6 H‐score 114.4 vs. 141.2 control; TNF‐α H‐score 47.8 vs. 101.4 control; CD206/IL‐1β ratio 5.24 vs. 1.14 control	DDMNS achieves programmed burst + sustained drug release, synergistically combining Mg‐triggered immune modulation and PNS‐driven angiogenesis, accelerating infected diabetic wound healing with enhanced neovascularisation and collagen deposition	Preclinical animal study; long‐term biosafety and large‐scale clinical translation require further validation
49	Pu et al. (2024)	China–type 2 diabetic rat full‐thickness dorsal wound model	Construction of nanozyme‐functionalised regenerative microenvironmental regulator (AHAMA/CS‐GOx@Zn‐POM): aldehyde and methacrylic anhydride–modified hyaluronic acid hydrogel (AHAMA) embedding chitosan nanoparticles loaded with glucose oxidase (GOx) and zinc‐based polyoxometalate nanozyme (Zn‐POM); in vitro antioxidant, angiogenesis, macrophage polarisation and in vivo diabetic wound healing assays	STZ‐induced type 2 diabetic Sprague–Dawley rats (10‐mm full‐thickness dorsal wounds); HUVECs; RAW264.7 macrophages; L929 fibroblasts	AHAMA/CS‐GOx@Zn‐POM hydrogel with UV‐crosslinkable aldehyde–methacrylic HA matrix and CS nanoparticles co‐loaded with GOx and Zn‐POM; GOx converts glucose to gluconic acid, Zn‐POM shows catalase and SOD‐like activities	GOx lowers glucose but generates H_2_O_2_; Zn‐POM scavenges ROS and H_2_O_2_ while releasing Zn^2+^ to activate pro‐healing pathways; Zn‐POM inhibits MAPK/IL‐17 signalling, ↓ TNF‐α/IL‐6/CD86, ↑ IL‐4, promoting M1 → M2 macrophage polarisation and enhancing angiogenesis/collagen deposition	Zn‐POM CAT and SOD activity: 27.9 U/mL and 46.4 U/mL; strong scavenging of H_2_O_2_, ABTS, hydroxyl, and superoxide radicals Optimal GOx:Zn‐POM ratio 1:10 balances glucose depletion and ROS clearance HUVEC migration ↑2.4‐fold vs. hyperglycemic control; tube formation ↑nodes and total length RAW264.7 M2 polarisation: CD206↑, CD86↓; ROS ↓ significantly Diabetic rats: wound closure ~90% by Day 14 vs. ~60% PBS; dense collagen (Col I), abundant CD31+ neovasculature, hair follicle regeneration	AHAMA/CS‐GOx@Zn‐POM hydrogel provides integrated regulation of hyperglycemic and immune microenvironments, combining glucose depletion with ROS scavenging to drive M2 macrophage polarisation, enhance angiogenesis and collagen regeneration, and markedly accelerate diabetic wound healing	Preclinical animal study: long‐term biosafety and clinical translation of nanozyme‐hydrogel system require further validation
50	Sawaya et al. (2022)	USA–human DFU biopsies, STZ‐induced and db/db diabetic mouse wound models	Mechanistic study of FOXM1–TREM1 network in neutrophil extracellular trap (NET) formation; human DFU vs. acute wound RNA‐seq; in vitro human neutrophil assays; in vivo TREM1 activation in diabetic mice	Human DFU tissues (*n* = 13) and acute wound biopsies (*n* = 8); human peripheral blood neutrophils; STZ‐induced diabetic mice; db/db mice (6‐mm full‐thickness dorsal wounds)	Not a biomaterial study (mechanistic investigation of immune signalling)	Loss of FOXM1 in DFUs increases ROS and NET formation; TREM1 activation recruits FOXM1^+^ neutrophils, inhibits NETs, and promotes wound healing	Human DFUs show suppressed FOXM1 and TREM1 vs. acute wounds FOXM1 inhibition (FDI‐6) ↑ ROS and NET formation in human neutrophils; NAC rescues effect TREM1 activation in diabetic mice ↑ FOXM1+ neutrophils, ↓ citH3+ NETs, accelerates wound closure at days 2–4 Healing DFUs express higher TREM1, CXCL8, CXCR1/2 and lower citH3 than nonhealing DFUs	TREM1 activation restores FOXM1‐mediated control of ROS, reduces NET formation and significantly accelerates diabetic wound healing	Mechanistic preclinical and translational study; requires clinical trials to confirm therapeutic potential of TREM1/FOXM1 targeting
51	Smith et al. (2021)	USA/3D human skin equivalent (HSE) disease model	Development of a 3D human skin equivalent (HSE) incorporating DFU‐derived fibroblasts and blood‐derived monocytes/macrophages from diabetic patients; in vitro inflammatory assays	DFU fibroblasts (*n* = 3) and site‐matched non‐diabetic fibroblasts (*n* = 3); PBMC‐derived monocytes from diabetic and control patients; neonatal human keratinocytes	Type I collagen‐based HSE seeded with DFU fibroblasts, patient‐derived monocytes/macrophages and keratinocytes	Monocytes differentiated in situ into macrophages with persistent M1 pro‐inflammatory phenotype, secreting elevated IL‐1β, IL‐6, IL‐8, TNF‐α; interaction between DFU fibroblasts and macrophages directs macrophage polarisation toward M1	Monocytes differentiated into macrophages within HSEs and secreted M1‐associated cytokines. DFU fibroblasts induced naive macrophages to express HLADR and maintain pro‐inflammatory cytokine secretion. HSEs reproduced chronic inflammatory DFU microenvironment, suitable for drug screening and mechanistic studies.	First human 3D skin model integrating patient‐derived macrophages and fibroblasts faithfully recapitulates DFU‐like chronic inflammation, providing a platform for preclinical drug testing	Small number of patient samples; lacks other immune cell types and vascularisation; further validation needed before clinical or high‐throughput application
52	Tan et al. (2021)	Australia–STZ‐induced diabetic Leprdb/db mouse full‐thickness wound model	Protein engineering of matrix‐binding interleukin‐1 receptor antagonist (IL‐1Ra/PlGF123–141) and PDGF‐BB/PlGF123–141; in vitro ECM‐binding and macrophage assays; in vivo diabetic wound healing, cytokine, MMP and fibroblast senescence analyses	Leprdb/db diabetic mice and IL‐1R1‐deficient Leprdb/db‐Il1r1−/− mice; human and mouse dermal fibroblasts; murine bone marrow–derived macrophages	Recombinant IL‐1Ra/PlGF123–141 fusion protein (super‐affinity IL‐1Ra) engineered to strongly bind extracellular matrix without biomaterial carrier	IL‐1Ra/PlGF123–141 blocks IL‐1R1 signalling, reduces IL‐1β–driven inflammation, accelerates neutrophil clearance and promotes M1 → M2 macrophage polarisation (↑CD206), ↓ pro‐inflammatory cytokines (IL‐1β, IL‐6, CXCL1), ↓ MMP‐2/9, ↑ anti‐inflammatory cytokines (TGF‐β1, IL‐4, IL‐10), ↑ TIMP‐1, ↑ pro‐healing growth factors (FGF‐2, PDGF‐BB, VEGF‐A); ↓ fibroblast senescence	Diabetic mice deficient in IL‐1R1 healed significantly faster than controls (near‐complete closure by Day 9 vs. ~50% open wounds) Single 0.5 μg dose of IL‐1Ra/PlGF123–141 accelerated re‐epithelialisation and angiogenesis (↑CD31+, desmin+ vessels) compared to wild‐type IL‐1Ra and PDGF‐BB Reduced SA‐β‐gal fibroblast senescence to levels of uninjured skin	Topical super‐affinity IL‐1Ra re‐establishes a pro‐healing microenvironment by dampening IL‐1R1 signalling, reducing chronic inflammation and fibroblast senescence, and enhancing angiogenesis, leading to rapid closure of diabetic wounds	Preclinical animal study; requires human clinical trials to confirm efficacy and safety of matrix‐binding IL‐1Ra therapy
53	Tang et al. (2023)	China–STZ‐induced diabetic Sprague–Dawley rat dorsal wound model	Preparation of temperature‐ and pH‐dual responsive injectable self‐healing hydrogel (FCAB) from Pluronic F127–chitosan oligosaccharide (F127‐COS), aldehyde hyaluronic acid (A‐HA), chitosan oligosaccharide (COS), and boric acid (BA), loaded with deferoxamine (DFO); in vitro angiogenesis assays; in vivo diabetic wound healing	STZ‐induced diabetic rats (8‐mm full‐thickness dorsal wounds); HUVECs; NIH3T3 fibroblasts; L929 fibroblasts	FCAB/DFO hydrogel: F127‐COS/A‐HA/COS/BA network, thermosensitive (gelation at 37°C), pH‐responsive (Schiff base bonds); DFO 0.4 μM for pro‐angiogenic therapy	DFO activates HIF‐1α signalling, ↑ VEGF and SDF‐1α; hydrogel's pH sensitivity enables faster DFO release at acidic pH; promotes HUVEC migration/tube formation and angiogenesis in vivo	pH 5.0: ~70% DFO released in 6 h vs. ~30% at pH 7.4/24 h > 90% 3T3 and L929 viability; good cytocompatibility HUVEC migration and tube formation significantly ↑ with FCAB/DFO Day‐14 wound closure 91.44% ±6.08 vs. < 65% controls Histology: dense collagen, hair follicles, sweat glands Immunohistochemistry: ↑ CD31 and α‐SMA indicating mature neovascularisation	FCAB/DFO hydrogel provides dual stimulus–responsive controlled DFO release, enhancing angiogenesis and significantly accelerating diabetic foot ulcer healing	Preclinical animal study; long‐term biosafety and molecular mechanism studies needed before clinical translation
54	Tang et al. (2025)	China–STZ‐induced type 1 diabetic mouse full‐thickness dorsal wound model	Development of dual‐action therapy: a wound microenvironment–responsive multifunctional hydrogel (GDHPC) plus tail‐vein injection of human umbilical cord MSC–derived exosomes (hucMSC‐exos); in vitro antibacterial, antioxidant, anti‐inflammatory, keratinocyte migration assays; in vivo glucose regulation and diabetic wound healing	STZ‐induced type 1 diabetic mice (8‐mm full‐thickness dorsal wounds); NIH/3T3 fibroblasts; L929 fibroblasts; HaCaT keratinocytes; human umbilical cord MSC–derived exosomes	GDHPC hydrogel: gelatin–dopamine (Gel‐DA) crosslinked with hyaluronic acid–phenylboronic acid (HA‐PBA), loaded with ciprofloxacin hydrochloride (CIP·H); injectable, self‐healing, adhesive; responsive to low pH, high glucose and ROS for on‐demand antibacterial CIP·H release	Dopamine and PBA groups scavenge ROS and reduce inflammation; GDHPC hydrogel ↓ IL‐6 and promotes HaCaT migration; hucMSC‐exos modulate pancreatic immune microenvironment, repair islet injury, ↑ insulin secretion and ↓ blood glucose; synergistic M1 → M2 macrophage polarisation (↓iNOS, ↑CD206) and enhanced angiogenesis (↑CD31)	Nearly 100% in vitro bacterial killing (E. coli, S. aureus) GDHPC hydrogel: ~100% H_2_O_2_ scavenging in 12 h; strong IL‐6 downregulation in LPS‐stimulated L929 HaCaT migration ↑ under high glucose Tail‐vein hucMSC‐exos ↓ blood glucose and ↑ serum insulin In vivo: GDHPC‐exo wounds 94.8% ± 4.5% closure by Day 14 vs. < 65% control; thick granulation tissue, dense collagen, complete re‐epithelialisation; IL‐6 and iNOS ↓, CD206 and CD31 ↑	Dual‐action strategy of GDHPC hydrogel + systemic hucMSC‐exos provides inside‐out repair by simultaneously improving local wound microenvironment and systemic glucose regulation, accelerating diabetic wound healing with enhanced angiogenesis and reduced inflammation	Preclinical animal study; requires long‐term biosafety evaluation and human clinical trials before translation
55	Tao et al. (2025)	China–STZ‐induced type 1 diabetic C57BL/6 mouse full‐thickness dorsal wound model	Investigation of interleukin‐27 (IL‐27) supplementation in diabetic wound healing; in vitro macrophage assays (RAW264.7 and bone marrow‐derived macrophages) under high glucose/LPS; in vivo IL‐27 treatment and macrophage‐specific IL‐27Rα knockout experiments	STZ‐induced diabetic C57BL/6 mice (6‐mm dorsal full‐thickness wounds); RAW264.7 cells; bone marrow–derived macrophages	No biomaterial—cytokine therapy with recombinant mouse IL‐27 (200 or 400 ng/wound intradermal injection)	IL‐27 binds IL‐27Rα, activates STAT3 phosphorylation, restores early M1 polarisation and promotes subsequent M1 → M2 macrophage transition, reducing chronic inflammation and enhancing angiogenesis	IL‐27 expression and IL‐27Rα levels ↓ in diabetic mouse skin vs. normal IL‐27 treatment (400 ng/wound) accelerated wound closure on Day 8 (~complete) vs. delayed healing in untreated DM mice Early (Day 2) ↑F4/80+ macrophage infiltration and ↑M1 markers (iNOS, IL‐1β) • Later (Day 8) ↓IL‐1β, ↑M2 markers (Arg‐1, CD206), ↑collagen deposition, ↑CD31+ neovascularisation IL‐27 rescued high glucose–induced suppression of macrophage migration, phagocytosis and M1 gene expression in vitro Macrophage‐specific IL‐27Rα knockout blocked STAT3 phosphorylation, impaired M1 → M2 transition and delayed wound healing	Exogenous IL‐27 restores IL‐27–IL‐27Rα–p‐STAT3 signalling, orchestrating sequential macrophage polarisation and markedly accelerating diabetic wound healing	Preclinical animal study: human DFU validation and long‐term safety of IL‐27 therapy are needed before clinical translation
56	Wang et al. (2025)	China–STZ‐induced diabetic C57BL/6 mouse full‐thickness dorsal wound model	Development of trehalose‐modified alginate–polyacrylamide “breathable hydrogel” (PATP) containing palladium hydride (PdH) nanocubes; in vitro antioxidant/anti‐inflammatory assays and in vivo diabetic foot ulcer (DFU) healing evaluation	STZ‐induced diabetic C57BL/6 mice (8‐mm full‐thickness dorsal wounds); RAW264.7 macrophages; HUVECs	PATP hydrogel: double crosslinked alginate–polyacrylamide network with trehalose; embedded PdH nanocubes (0.05 wt%) providing catalase‐ and SOD‐like activity and NIR‐triggered H_2_ release	“Head and tail” co‐blocking of HMGB1‐RAGE axis: PdH nanozyme catalytically scavenges ROS to inhibit HMGB1 secretion (“head”), while H_2_ released suppresses RAGE expression (“tail”), breaking the inflammatory cascade and normalising immune microenvironment	PdH nanocubes show CAT/SOD‐like ROS scavenging (~75% H_2_O_2_, ~60% O_2_•−removal) and NIR‐induced H_2_ release (≈12.5 μM) RAW264.7 viability > 90%; intracellular ROS ↓; HMGB1 secretion ↓ to 4.7 ng/mL; RAGE expression ↓ to 6.67 ng Diabetic mice: wound closure 92.7% by Day 10 (vs. ~55% control); dense collagen, hair follicle regeneration; HMGB1 and RAGE immunofluorescence ↓ ~75%; TNF‐α expression ↓ ~50%; CD206+ M2 macrophages ↑; VEGF expression comparable to normal	PATP hydrogel provides dual HMGB1‐RAGE axis inhibition, combining ROS scavenging and H_2_‐mediated RAGE suppression to restore immune balance, enhance angiogenesis and collagen deposition, and markedly accelerate DFU healing	Preclinical animal study; long‐term biosafety of PdH nanocubes and clinical translation require further validation
57	Wu et al. (2025)	China–STZ‐induced diabetic C57BL/6 mouse full‐thickness dorsal wound model	Genetic engineering of human umbilical cord MSCs (hUMSCs) to simultaneously overexpress IL‐4, IL‐10 and IL‐13 (MSCs‐3IL); in vitro macrophage polarisation assays and multiple in vivo inflammatory disease models	hUMSCs; RAW264.7 macrophages; STZ‐induced diabetic C57BL/6 mice (8‐mm full‐thickness wounds); additional rat/mouse models of vaginitis, osteoarthritis, acute pleurisy, colitis, pneumonia	No biomaterial scaffold—cell therapy using lentiviral‐modified hUMSCs (MSCs‐3IL)	Overexpression of IL‐4/IL‐10/IL‐13 activates STAT6 and NF‐κB inhibition, strongly promoting M1 → M2 macrophage polarisation (↓CD86, ↑CD206), reducing pro‐inflammatory cytokines (IL‐1β, IL‐6, TNF‐α) and enhancing re‐epithelialisation and angiogenesis	IL‐4 secretion ↑ to ~400 ng/mL; IL‐10 to ~200 ng/mL; IL‐13 to ~6 ng/mL RAW264.7 cells: IL‐1β, IL‐6, TNF‐α ↓; IL‐10, IL‐13, Arg1 ↑; CD163 (M2 marker) ↑ to 19% vs. 6% control Diabetic mice: wound closure > 96% by Day 14; ↑PCNA, CD31, F4/80; ↓CD86, ↑CD206 No karyotype changes or tumour formation in NOD‐SCID mice; luciferase imaging confirmed transient cell persistence	MSCs‐3IL therapy provides potent multi‐cytokine anti‐inflammatory action, driving macrophage reprogramming and markedly accelerating diabetic wound healing and repair in diverse inflammatory models	Preclinical animal study; long‐term safety, dosing, and efficacy in human DFU patients require further clinical validation
58	Xin et al. (2021)	China–db/db diabetic mouse full‐thickness dorsal wound model	Evaluation of human foreskin‐derived dermal stem/progenitor cell–conditioned medium (hFDSPC‐CM) combined with hyaluronic acid (HA) hydrogel; in vitro keratinocyte/fibroblast/ECM assays and in vivo diabetic wound healing	db/db type II diabetic mice (6‐mm full‐thickness dorsal wounds); HaCaT keratinocytes; human dermal fibroblasts; HUVECs	HA hydrogel enriched with hFDSPC‐CM (100 μg/mL); compared to hADSC‐CM + HA and HA alone	hFDSPC‐CM activates TGF‐β/Smad signalling, ↑ TGF‐β1 secretion, ↑ fibroblast myofibroblast differentiation (↑α‐SMA), ↓ MMP‐1/3, and promotes M1 → M2 macrophage polarisation (↓CD86, ↑CD206) reducing inflammation	Wound closure time: 13.8 ± 0.84 days vs. 22.6 ± 1.14 (control) ↑ complete re‐epithelialisation and collagen I/III ratio (2.63 ± 0.19 vs. < 0.5 control) • CD31+ microvessels ↑; macrophage infiltration (CD68+) ↓; CD86+ M1 ↓, CD206+ M2 ↑ HaCaT and fibroblast proliferation and migration significantly ↑; ECM proteins (COL1, COL3, FN) ↑; α‐SMA ↑; TGF‐β1 and p‐Smad2/3 ↑	hFDSPC‐CM combined with HA accelerates diabetic wound closure by enhancing keratinocyte/fibroblast proliferation, ECM deposition and remodelling, stimulating TGF‐β/Smad‐mediated collagen synthesis and shifting macrophages toward a pro‐healing M2 phenotype	Preclinical animal study; active factors in CM not fully identified and long‐term safety/immunogenicity require further investigation
59	Zhang et al. (2023)	China/STZ‐induced diabetic rat model; in vitro L929 fibroblasts, HUVECs, bone marrow‐derived macrophages (BMDMs)	Development of self‐healing, injectable chitosan‐based macromolecular hydrogel (CEC‐HA‐ADH) loaded with total glycosides of paeony (TGP); in vitro fibroblast/endothelial cell assays and in vivo STZ‐induced diabetic rat full‐thickness dorsal wound model	STZ‐induced diabetic rats (10‐mm dorsal wounds); L929 fibroblasts; HUVECs; bone marrow‐derived macrophages (BMDMs)	TGP@CEC‐HA hydrogel: N‐carboxyethyl chitosan (CEC), hyaluronic acid‐aldehyde (HA‐ALD), and adipic acid dihydrazide (ADH) crosslinked hydrogel delivering TGP	TGP exhibits ROS‐scavenging capacity and drives M1 → M2 macrophage polarisation; ↓ pro‐inflammatory cytokines (TNF‐α, IL‐1β, IL‐6), ↑ anti‐inflammatory cytokines (IL‐4, IL‐10, TGF‐β); ↑ fibroblast ECM protein secretion; ↑ HUVEC angiogenesis	56% H_2_O_2_ scavenged within 60 min; near‐complete ROS depletion within 120 min • Intracellular ROS markedly reduced in L929 and HUVECs Fibroblast proliferation, COL‐I/III, FN, EGF, FGF, VEGF expression ↑ HUVEC migration, tube formation ↑ under oxidative stress M1 macrophages (iNOS+) ↓, M2 (CD163+) ↑; IL‐4, IL‐10, TGF‐β ↑; TNF‐α, IL‐1β, IL‐6 ↓ • Day‐16 wound closure nearly complete vs. 55% control	TGP@CEC‐HA hydrogel regulates oxidative stress microenvironment, promotes fibroblast/ECM activity, induces M2 macrophage polarisation, enhances angiogenesis and collagen deposition, significantly accelerating diabetic wound healing	Preclinical animal study; requires systematic biosafety evaluation and large‐scale clinical validation before translation
60	Zhang et al. (2024)	China–STZ‐induced diabetic Sprague–Dawley rat infected full‐thickness dorsal wound model	Development of pH/glucose dual‐responsive supramolecular G‐quadruplex hydrogel (GBC) composed of guanosine, benzene‐1,4‐diboric acid (BDBA), and chlorogenic acid (CA); in vitro antibacterial, antioxidant, angiogenesis and macrophage polarisation assays; in vivo diabetic wound healing	STZ‐induced diabetic SD rats with *S. aureus* ‐infected dorsal wounds; NIH3T3 fibroblasts; HUVECs	GBC hydrogel: guanosine‐phenylboronic‐chlorogenic acid network crosslinked by borate bonds, forming a potassium ion–stabilised G‐quadruplex; releases CA under acidic and high‐glucose conditions	CA provides ROS scavenging and antibacterial activity; borate bonds give pH/glucose‐responsive CA release; hydrogel promotes M1 → M2 macrophage polarisation (↓CD86, ↑CD206), ↓ TNF‐α, ↑ IL‐10, and enhances VEGF/CD31/α‐SMA expression	Dual‐responsive CA release: > 70% at pH 6.5 in 12 h; > 50% with high glucose • Potent antibacterial activity vs. *S. aureus* and *E. coli* (live/dead staining and inhibition zones) Strong ABTS+ radical scavenging and ROS reduction in NIH3T3 cells HUVEC migration/tube formation ↑; VEGF and CD31 ↑ In diabetic infected wounds: bacterial load ↓, collagen deposition ↑, hair follicles and blood vessels ↑; CD206 ↑, CD86 ↓; IL‐10 ↑, TNF‐α ↓; VEGF, CD31, α‐SMA ↑; nearly complete wound closure by Day 14	GBC hydrogel provides integrated antibacterial, antioxidant, anti‐inflammatory and pro‐angiogenic therapy, accelerating healing of infected diabetic foot ulcers	Preclinical animal study; requires long‐term biosafety evaluation and clinical trials before translation
61	Zhai et al. (2025)	China–STZ‐induced type 1 diabetic C57BL/6 mouse full‐thickness dorsal wound model	Construction of Gelatin Methacryloyl (GelMA) hydrogel loaded with interleukin‐4 (IL‐4)–engineered PD‐L1–enriched exosomes (ExosIL‐4); in vitro HUVEC migration/tube formation and macrophage polarisation assays; in vivo diabetic wound healing	STZ‐induced diabetic C57BL/6 mice (1 cm full‐thickness dorsal wounds); HUVECs; NIH3T3 fibroblasts (source of Exos)	GelMA/ExosIL‐4 hydrogel: light‐curable 15% GelMA hydrogel encapsulating IL‐4–overexpressing NIH3T3 fibroblast‐derived exosomes enriched in PD‐L1	Exosomal PD‐L1 binds PD‐1 on T cells, suppressing T cell activation; IL‐4 overexpression promotes M2 macrophage polarisation (↓iNOS, ↑CD206) and reduces chronic inflammation while enhancing angiogenesis	Sustained exosome release up to 7 days; strong injectability, adhesion (~1.2 kPa) and self‐healing HUVEC viability maintained; migration and tube formation ↑ under high glucose Diabetic mice: wound closure nearly complete by Day 10 vs. 10%–15% open area in controls; ↑ collagen I/III, CK14, CD31; ↓ CD4+/CD8+ T cells, ↓ iNOS, ↑ CD206	GelMA/ExosIL‐4 hydrogel provides dual immunomodulation and pro‐angiogenic effects, accelerating diabetic wound healing by promoting M2 macrophage polarisation, inhibiting T cell proliferation, and enhancing angiogenesis and re‐epithelialisation	Preclinical animal study; long‐term safety and clinical translation of PD‐L1–exosome hydrogel therapy require further validation
62	Zhao et al. (2023)	China–STZ‐induced diabetic Sprague–Dawley rat full‐thickness dorsal wound model	Development of biomimetic nanozyme‐decorated conductive hydrogel (MnCoO@PDA/CPH) with H_2_O_2_‐activated oxygenation; in vitro skin cell assays; in vivo diabetic wound healing	STZ‐induced diabetic rats (full‐thickness dorsal wounds); human keratinocytes (HaCaT), human dermal fibroblasts (HDFs), human arterial endothelial cells (HAECs), RAW264.7 macrophages	MnCoO@PDA/CPH hydrogel: polyvinyl alcohol (PVA), hyaluronic acid (HA), aniline (AN), 3‐aminophenylboronic acid (ABA), polydopamine (PDA)‐coated MnCoO nanozyme, polyaniline‐based conductive hydrogel	CAT‐mimic MnCoO@PDA nanozyme catalytically scavenges H_2_O_2_ to generate O_2_, relieving hypoxia and oxidative stress; reduces M1 (CD86^+^) and promotes M2 (CD163^+^) macrophage polarisation; ↓ TNF‐α, IL‐1β, ↑ IL‐10, TGF‐β	> 65% H_2_O_2_ removed in 10 min; O_2_ increased from 7.03 to 15.30 mg/L Keratinocyte, fibroblast, endothelial cell proliferation/migration ↑ under ROS stress RAW264.7: NO ↓ from 49.2 to 10.6 μM; TNF‐α and IL‐1β ↓; IL‐10 and TGF‐β ↑ Diabetic rats: nearly complete wound closure by Day 16 vs. > 50% residual in controls; improved granulation tissue, collagen deposition, re‐epithelialisation, and angiogenesis	MnCoO@PDA/CPH hydrogel provides simultaneous ROS scavenging and oxygenation, normalising the immune microenvironment, promoting M1 → M2 macrophage transition, and markedly accelerating diabetic wound healing	Preclinical animal study; requires long‐term biosafety assessment and human clinical validation before clinical translation
63	Zhou et al. (2025)	China–integrated multi‐omics and experimental validation	Integrated bioinformatics and multi‐omics analysis (GEO datasets GSE60436, GSE80178, GSE134431), machine learning (LASSO, SVM, Random Forest), WGCNA, single‐cell RNA sequencing, molecular docking, Mendelian randomisation, qRT‐PCR, and immunohistochemistry to identify ER stress biomarkers in diabetic foot ulcers	Human DFU tissue samples (*n* = 5) and normal tissue controls (*n* = 5); GEO transcriptomic datasets; HUVECs cultured under high glucose	No biomaterial scaffolding‐in silico biomarker and therapeutic target discovery	Identified KDELR3 and YOD1 as key ER stress (ERS) proteins; metronidazole predicted as a small‐molecule inhibitor via molecular docking; IL‐1β, TNF‐α pathways implicated; immune cell infiltration analysis showed correlations of ERRGs with M1/M2 macrophage balance	32 differentially expressed ERS‐related genes in DFU; 4 hub genes (BOK, KDELR3, NCCRP1, YOD1) selected by machine learning KDELR3 and YOD1 validated as diagnostic biomarkers (AUC 0.875 and 0.856 in external validation) Metronidazole docking energy < −5.0 kcal/mol to KDELR3 and YOD1; in vitro high‐glucose model: metronidazole significantly downregulated YOD1, KDELR3, BOK expression Immune infiltration: KDELR3 positively correlated with M1 macrophages; YOD1 negatively correlated with M2 macrophages	KDELR3 and YOD1 are novel ERS biomarkers with strong diagnostic potential; metronidazole is a promising therapeutic candidate targeting these proteins; findings improve understanding of ER stress–immune microenvironment interactions in DFU	Observational bioinformatics and preclinical validation; findings limited by small human sample size, population heterogeneity, and need for clinical validation of metronidazole's therapeutic effect
64	Zhou et al. (2025)	China–db/db type 2 diabetic mouse full‐thickness dorsal wound model	Development and evaluation of Lactobacillus biofilm derivatives (LBDs)—bacteria‐free biofilm therapy; in vitro macrophage polarisation assays and in vivo diabetic wound healing; transcriptomics and metabolomics	db/db diabetic mice (7 mm full‐thickness dorsal wounds); RAW264.7 macrophages; NIH3T3 fibroblasts	LBDs: Lactobacillus biofilm derivatives obtained by ultrasonic separation and filtration of *Lactobacillus reuteri* biofilms grown on inverse opal films; bacteria‐free extract retaining bioactive components	LBDs inhibit JAK/STAT1 signalling, reprogram macrophage metabolism toward oxidative pathways, promote M1 → M2 macrophage polarisation (↓iNOS, ↑CD206, ↑Arg‐1), alleviate local inflammation and enhance neovascularization	Day‐14 wound closure significantly ↑ with LBDs vs. PBS control CD206+ macrophages ↑, iNOS+ ↓ on days 4 and 7 • IL‐6 and IL‐1β ↓, IL‐10 ↑ in wound tissue and macrophages JAK1 and p‐STAT1 protein levels ↓ in vitro and in vivo; IL‐6 agonist experiments confirmed pathway inhibition Transcriptomics/metabolomics: ↓ TCA cycle and electron transport chain activity; ↑ glutathione metabolism; shift to oxidative metabolism consistent with M2 phenotype	LBDs provide a natural, safe, bacteria‐free probiotic therapy, accelerating diabetic wound healing by metabolically reprogramming macrophages and suppressing JAK/STAT1‐driven inflammation	Preclinical animal study; long‐term biosafety, identification of active LBD components, and clinical validation needed

### 2.5 Assessment of Methodological Quality

Risk of bias and overall methodological quality were independently assessed by two reviewers using validated tools appropriate to each study design:

- *Preclinical animal studies*: evaluated with the *SYRCLE Risk of Bias tool*, which assesses randomisation (sequence generation, allocation concealment), blinding of caregivers and outcome assessors, completeness of outcome data, selective outcome reporting, and other potential sources of bias [70].
- *In vitro mechanistic studies*: assessed with an *adapted checklist* based on the OHAT risk‐of‐bias guidance, focusing on reproducibility of experimental procedures, control of confounding variables, and clarity of outcome measurement [71].
- *Clinical studies*: evaluated using the *Joanna Briggs Institute (JBI) critical appraisal checklists* appropriate to the study design (e.g., case series or pilot clinical trials) [72].

Each item was rated as “low risk,” “high risk” or “unclear risk.” Disagreements were resolved by discussion or consultation with a third reviewer. A qualitative summary of these assessments is presented in Table S2; no study was excluded solely on the basis of quality.

### 2.6 Risk‐of‐Bias Visualisation

Risk‐of‐bias assessments were summarised graphically using the *robvis* web application [73]. For each study, domain‐level judgements from the SYRCLE tool (animal experiments), the OHAT framework (in vitro studies), and, where applicable, the JBI checklist (pilot human case series) were entered into robvis. The tool generated: Traffic‐light plots showing risk‐of‐bias judgements (“Low,” “Moderate/Some concerns,” “High” or “Unclear”) for each domain across individual studies, and weighted summary bar plots showing the proportion of studies rated at each risk level for each domain.

These figures appear in the Section 3 and in the Supporting Information: Figures, providing an at‐a‐glance overview of methodological quality.

### 2.7 Ethical Considerations

This review synthesises data from previously published studies and did not involve the collection of new human or animal data. All included primary studies reported obtaining appropriate institutional ethics approval and informed consent when applicable. Because the present review analyses data already in the public domain, formal ethical approval or informed consent was not required, in accordance with the Declaration of Helsinki and local institutional policies.

## 3 Results

### 3.1 Characteristics of the Studies

The 34 studies included in this systematic review (Table 1) primarily employed preclinical animal models, such as streptozotocin (STZ)‐induced diabetic rats/mice and db/db mice. A smaller subset incorporated in vitro mechanistic experiments (e.g., HUVEC, RAW264.7, BMDM, HaCaT cell lines) and only limited clinical or pilot data. Two notable human‐based examples are the 3D human skin equivalent (HSE) created with patient‐derived cells [51] and the natural ECM‐hydrogel approach reporting the first‐in‐human pilot application in DFU patients [35]. Most of the remaining studies evaluated the effects of immunomodulation on regenerative outcomes in full‐thickness wound repair using diabetic animal models [32, 33, 34, 36, 37, 38, 39, 40, 42, 44, 45, 46, 47, 48, 49, 50, 52, 53, 54, 55, 56, 58, 59, 61, 62, 63, 64].

Biomaterials described in these studies were predominantly natural polymer–based hydrogels, especially those incorporating hyaluronic acid (HA), gelatin/collagen, and chitosan derivatives, often combined with antioxidant compounds (e.g., bilirubin complexes, plant‐derived small molecules), immune‐modulating cytokines (IL‐4, IL‐10, IL‐13, IL‐27), or exosomes [36, 40, 55, 57, 58, 59, 61]. Among synthetic or hybrid platforms, notable examples include electrospun nanofiber membranes (PCL/gelatin), conductive or nanozyme‐loaded hydrogels, and microneedle systems, as well as portable electrospun dressings and in situ production strategies [42, 45, 48, 49, 62]. More advanced designs—such as dynamic networks capable of autonomously adjusting viscoelasticity, oxygen‐releasing/photosynthetic dressings, and antioxidant–antimicrobial composites containing metal nanocubes or zero‐valent selenium‐ aim to reprogram the DFU microenvironment in a multi‐axial fashion [34, 36, 46, 47, 56].

Immunomodulatory strategies predominantly targeted macrophage polarisation, promoting the shift from the pro‐inflammatory M1 to the anti‐inflammatory M2 phenotype. This was achieved through diverse mechanisms: cytokine administration (e.g., MSCs secreting IL‐4/IL‐10/IL‐13; IL‐27 delivery), alleviation of oxidative stress or hypoxia (nanozymes, oxygen‐producing systems, antioxidant small molecules), metabolic reprogramming (e.g., glycolysis restriction, selenoprotein synthesis), cell‐derived signals (MSC exosomes), and electrical or microcurrent stimulation [35, 43, 46, 49, 55, 57, 61, 62]. These interventions consistently led to resolution of chronic inflammation, enhanced angiogenesis, accelerated wound closure, improved collagen maturation, and faster epithelialisation. In infection‐containing submodels, bioactive or antimicrobial designs—including phenylboronic acid–containing capture‐kill HA hydrogels, GBC hydrogels, Ppy‐hybrid photothermal hydrogels, and sericin–rGO–teicoplanin hybrids—provided simultaneous immune rebalancing and bacterial control [34, 37, 44, 60].

### 3.2 Discovery of New Biomarkers Using Multi‐Omic and Bioinformatics Approaches

Zhou et al. performed multi‐omic and bioinformatics analyses of GEO datasets to identify biomarkers associated with DFU‐related endoplasmic reticulum (ER) stress [63]. Using WGCNA and machine‐learning methods, they identified KDELR3 and YOD1 as biomarkers with high diagnostic value (AUC 0.875 and 0.856, respectively) and highlighted metronidazole as a potential inhibitor. In high‐glucose models, metronidazole reduced expression of both genes. Moreover, KDELR3 correlated with M1 macrophages and YOD1 with M2 macrophages (Table 1).

### 3.3 Cytokine‐ and Cell‐Based Immunomodulation

Tao et al. activated the IL‐27–IL‐27Rα–pSTAT3 axis with recombinant IL‐27, inducing early‐phase M1 polarisation and late‐phase M2 polarisation to accelerate DFU healing [55]. Human umbilical cord–derived mesenchymal stem cells (hUMSCs) engineered to overexpress IL‐4/IL‐10/IL‐13 promoted a robust M1 → M2 transition and enhanced angiogenesis [57]. Bai et al. reported that a self‐healing chitosan/HA hydrogel loaded with BM‐MSCs reduced chronic inflammation and supported tissue repair by increasing the M2 macrophage ratio (Table 1) [33].

### 3.4 Exosomes and Conditioned Medium

Zhai et al. demonstrated that a GelMA hydrogel containing IL‐4‐ and PD‐L1‐rich exosomes simultaneously induced M2 macrophage polarisation and suppressed T‐cell proliferation, accelerating DFU healing [61]. Xin et al. showed that an HA hydrogel enriched with hFDSPC‐conditioned medium (CM) activated the TGF‐β/Smad pathway, supported ECM remodelling, and reduced inflammation by promoting M2 polarisation (Table 1) [58].

### 3.5 Antioxidant and ROS‐Targeted Nanozymes

Pu et al. combined glucose consumption with CAT/SOD‐like ROS scavenging in a GOx + Zn‐POM hydrogel, enhancing M2 polarisation and angiogenesis [49]. Zhao et al. normalised hypoxia and oxidative stress via H_2_O_2_‐activated oxygenation using a conductive hydrogel with MnCoO@PDA nanozymes, thereby modulating the immune microenvironment [62]. Chen et al. achieved ROS elimination and M2 repolarisation with a dynamic HA hydrogel releasing H_2_S in coordination with Zn^2+^ [36]. Wang et al. bidirectionally blocked the HMGB1–RAGE axis and reduced inflammation through ROS scavenging and NIR‐triggered H_2_ release using a PdH nanocube‐loaded hydrogel [56]. Liu et al. triggered in situ selenoprotein synthesis with zero‐valent selenium nanoparticles, suppressed NF‐κB signalling, and increased the M2 macrophage ratio (Table 1) [46].

### 3.6 Natural Polymer Hydrogels

Zhang et al. showed that TGP@CEC‐HA hydrogel provided strong ROS scavenging, induced M1 → M2 polarisation, and significantly increased angiogenesis [59]. Fu et al. used protocatechuic aldehyde in an FGMA/FG/PA bioadhesive hydrogel, highlighting antibacterial effects and M2 orientation [12]. Hauck et al. demonstrated that an HA‐AC/collagen hydrogel releasing highly sulfated HA suppressed TLR/NF‐κB/STAT1 signalling and resolved inflammation [41]. Geng et al. reported that a puerarin–borax–PVA hydrogel downregulated JAK–STAT/Tnf, activated M2 macrophages, and promoted angiogenesis and collagen maturation [38]. Mai et al. enhanced JAK/STAT‐mediated M2 polarisation and VEGF production by modulating only the viscoelasticity of a dynamic LZM‐HZ hydrogel [47]. Tang et al. increased angiogenesis by activating the HIF‐1α–VEGF/SDF‐1α pathway using a pH/heat‐sensitive FCAB/DFO hydrogel [53]. He et al. accelerated M2 polarisation and re‐epithelialisation through KEAP1/NRF2 activation and NF‐κB suppression with a PCL/gelatin + CS‐4‐Octyl itaconate nanofiber membrane (Table 1) [42].

### 3.7 Combined Immunomodulation for Antibacterial and Infected DFU Models

Lan et al. achieved bacterial capture and killing with a HAMA‐AAPBA + QCS (HAQ) hydrogel, reducing TNF‐α levels and increasing CD31‐mediated angiogenesis [44]. A guanosine‐phenylboronic‐chlorogenic acid (GBC) hydrogel provided pH/glucose‐responsive chlorogenic acid release, antioxidant and antibacterial effects, and M2 polarisation [60]. Chu et al. employed a Ppy/PVA/PEG/HA hybrid hydrogel activated by 808 nm NIR photothermal stimulation to rapidly clear infection via early M1 activation and complement enhancement, followed by M2 transition and accelerated tissue repair [37]. Bakadia et al. reported strong antibiofilm activity and M2 polarisation using a teicoplanin‐decorated reduced graphene oxide–silk protein hybrid hydrogel, which protected bone tissue in a diabetic osteomyelitis model (Table 1) [34].

### 3.8 Electrospinning, Nanofibers, and Portable Dressings

Alizadeh et al. integrated 0.8% CuO nanoparticles into a PCL/SSA‐PVA core–shell nanofiber scaffold, enabling controlled Cu^2+^ release and upregulating VEGFA and bFGF, which accelerated DFU healing [32]. Li et al. developed a portable electrospun PED4 nanofiber dressing (PVB/Pluronic + kaempferol) that suppressed MMP9 and NF‐κB signalling, enhanced M2 polarisation and angiogenesis, and promoted rapid wound closure [45]. Similar antioxidant and immunomodulatory effects were observed with an electrospun nanofiber membrane (Table 1) [42].

### 3.9 Electrical/Microcurrent Stimulation

Kim et al. designed a self‐microcurrent‐generating dressing (“M‐sheet”) composed of PVA/gelatin nanofibers coated on a Zn/AgCl redox membrane. This microcurrent stimulation supported M1 → M2 polarisation, accelerated angiogenesis and tissue maturation, and enhanced DFU healing (Table 1) [43].

### 3.10 Microneedles/Controlled Release Systems

Ning et al. created a double‐layer detachable microneedle (DDMNS) system providing rapid burst release from the Mg‐PVP layer and sustained release from PNS‐chitosan tips. This two‐stage release significantly reduced inflammation and enhanced angiogenesis, accelerating healing in infected diabetic wounds (Table 1) [48].

### 3.11 Metabolic Reprogramming and Signalling Pathway Targeting

Chen et al. used an NPV‐ECM hydrogel to enhance M2 polarisation and angiogenesis via a “hyperinflammatory starvation” strategy that restricts macrophage glycolysis; a pilot trial in six DFU patients confirmed accelerated wound healing [35]. Zhou et al. reduced inflammation and induced M2 metabolism by inhibiting the JAK/STAT1 pathway with bacteria‐free Lactobacillus biofilm derivatives (LBDs) [64]. Tan et al. decreased chronic inflammation and fibroblast senescence by blocking IL‐1R1 signalling with matrix‐bound IL‐1Ra [52]. Sawaya et al. found that TREM1 activation increased FOXM1(+) neutrophil recruitment and suppressed NET formation, thereby accelerating DFU healing (Table 1) [50].

### 3.12 Angiogenesis‐Focused Small Molecule/Ion Strategies

Tang et al. triggered HIF‐1α activation with a pH/heat‐sensitive FCAB hydrogel loaded with deferoxamine (DFO), increasing VEGF and SDF‐1α release and accelerating mature vessel formation [53]. Alizadeh et al. reported that CuO nanoparticles in a core–shell PCL/SSA‐PVA nanofiber scaffold enhanced CD31^+^ vessel density and re‐epithelialisation by upregulating VEGFA and bFGF through controlled Cu^2+^ release [32]. Guo et al. induced M2 polarisation via mannose receptor targeting with PPD‐liposomes loaded onto BSPMA microspheres, enhancing angiogenesis through activation of PI3K/Akt/mTOR and Raf/MEK/ERK signalling [39]. Hao et al. combined platelet‐derived trophic factors and NIR‐induced heat shock proteins in a GelAlg@rGO‐pEV photothermal hydrogel, promoting angiogenesis and new vessel formation (Table 1) [40].

### 3.13 Skin Equivalent/Human Cell‐Based Model

Smith et al. developed a 3D HSE model using DFU patient fibroblasts and macrophages derived from diabetic patient monocytes. Macrophages in this model exhibited chronic inflammation with a persistent M1 phenotype, providing an innovative preclinical platform for drug screening and mechanistic studies of DFU treatments (Table 1) [51].

## 4 Risk of Bias

### 4.1 Animal Studies (SYRCLE)

Randomisation (sequence generation) and allocation concealment were rarely reported and thus frequently judged as **“**uncertain risk.” Studies that documented similar baseline characteristics (e.g., weight, age, sex) between groups were rated “low risk.” Random housing and blinding were generally unreported and scored “uncertain risk,” as were random outcome assessment and assessor blinding. Missing data and selective reporting were generally well managed and rated “low risk.” No obvious systematic issues were identified, and most studies provided transparent funding and conflict‐of‐interest statements (Table S2).

### 4.2 OHAT Adaptation (In Vitro Studies)

Cell source, passage, dose/time point, and sample‐size rationale were usually well described; however, pre‐experimental power analysis and protocol preregistration were rare. Load‐matched controls (e.g., empty hydrogel vs. bioactive‐loaded), high‐glucose/normal‐glucose conditions, and appropriate positive/negative controls were common, but blinded assessment was seldom reported. Validated methods for measuring cytokines, signalling pathways, ROS, and phenotypic markers were preferred, although prioritisation for multiple endpoints was rarely specified. Overall, in vitro studies were methodologically sound, but some domains remained at **“**uncertain risk**”** due to incomplete reporting (Table S2).

### 4.3 Pilot Human Data (JBI)

Sample and inclusion/exclusion criteria were clearly described; follow‐up and outcomes were standardised. However, small sample size and lack of a control group introduced potential selection and confounding bias [35]. No serious adverse events were reported, but long‐term safety and generalizability remain limited. The overall judgement was “some concerns/moderate risk.”

## 5 Discussion

Preclinical evidence in this systematic review is dominated by streptozotocin (STZ)‐induced diabetic rat/mouse and db/db mouse models, which underpin the vast majority of the 34 included studies [12, 32, 33, 34, 36, 37, 38, 39, 40, 41, 42, 43, 44, 45, 46, 47, 48, 49, 50, 52, 53, 54, 55, 56, 57, 58, 59, 60, 61, 62, 63, 64]. These models recapitulate salient DFU features—chronic inflammation, hypoxia, delayed angiogenesis—yet cannot fully capture the complexity of human immunity. Human‐based validation therefore remains essential for clinical translation.

Only a few studies incorporate human data, revealing a critical evidence gap. A 3D HSE created with patient‐derived cells reproduces DFU‐like chronic inflammation in vitro [51], and a first‐in‐human pilot of a natural ECM hydrogel provides encouraging feasibility signals [35]. Such platforms bridge animal findings to human immunobiology and enable early safety/performance assessment of novel immunomodulators.

Biomaterial designs are largely natural polymer–based hydrogels (hyaluronic acid, gelatin/collagen, chitosan) enriched with antioxidant small molecules, cytokines (IL‐4/IL‐10/IL‐13, IL‐27), or exosomes, which collectively suppress chronic inflammation and bias macrophages toward M2 polarisation [12, 37, 40, 55, 57, 58, 59, 61]. Alongside these, hybrid/synthetic platforms—electrospun nanofiber membranes, nanozyme‐loaded conductive hydrogels, microneedle systems, and portable electrospun dressings—are increasingly reported, particularly for infected DFU models [42, 45, 48, 49, 62]. This diversity enables multi‐axis microenvironment reprogramming through oxygenation, ROS scavenging, metabolic rewiring, and electrical stimulation [34, 36, 46, 47, 56].

A common mechanistic denominator is the M1 → M2 macrophage shift, achieved by cytokines [55, 57], oxidative‐stress alleviation [49, 62], metabolic reprogramming [35, 46], cell/exosome delivery [61] and electrical or microcurrent stimulation [43]. These strategies consistently resolve chronic inflammation, enhance angiogenesis, and accelerate re‐epithelialisation. In infected models, phenylboronic acid–containing HAQ hydrogels [44], GBC hydrogels [60], NIR‐stimulated Ppy hybrids [37] and teicoplanin–rGO–silk composites [34] couple antibacterial activity with immune modulation, further expediting repair.

Multi‐omics work expands the therapeutic toolkit. Zhou et al. identified ER‐stress–related biomarkers (KDELR3, YOD1) with high diagnostic value (AUC 0.875 and 0.856) and proposed metronidazole as a candidate inhibitor, which reduced gene expression in high‐glucose models [63]. The association of KDELR3 with M1 and YOD1 with M2 macrophages suggests a path toward molecular diagnosis and drug repositioning in DFU.

Cytokine/cell‐based approaches [33, 55, 57] directly induce M1 → M2 polarisation, boosting angiogenesis and tissue repair—supporting therapeutic immunomodulation as a means to overcome refractory inflammation. Exosome/conditioned‐medium strategies [58, 61] drive M2 polarisation and ECM remodelling, offering a potentially safer, more standardisable alternative to cell therapies for persistent inflammatory niches.

Across numerous studies, ROS‐targeted nanozymes, gas‐releasing hydrogels, and selenoprotein induction suppress inflammation and promote angiogenesis [36, 46, 49, 56, 62]. Given the centrality of hypoxia/oxidative stress in DFU, such multimodal antioxidant designs may complement standard care to yield faster, more durable closure.

Natural‐polymer hydrogels augmented with small molecules or ions reduce inflammation, curb oxidative stress, and stimulate angiogenesis, functioning as active biological therapeutics rather than passive scaffolds [12, 38, 41, 42, 47, 53, 59]. These platforms show promise as regenerative–immunomodulatory treatments that transcend conventional dressings.

Hybrid anti‐infective designs deliver dual benefits—pathogen control and immune rebalancing—thereby addressing antimicrobial resistance while advancing repair [34, 37, 44, 60]. Their combined action makes them attractive for DFU cases complicated by chronic, drug‐resistant infections.

Advances in electrospinning, portable dressings, and microcurrent‐generating redox membranes modulate macrophage responses through electrical, chemical, or mechanical cues and enable on‐the‐move, personalised care [32, 43, 45]. Microneedle‐based programmed delivery offers staged release that sequentially suppresses inflammation and fosters angiogenesis in infected wounds—capabilities beyond conventional dressings [48].

Targeting JAK/STAT, NF‐κB and HMGB1–RAGE pathways facilitates M1 → M2 transition and augments angiogenesis [35, 50, 52, 64]. Such molecularly targeted strategies complement standard wound care and lay groundwork for future clinical translation.

While macrophage polarisation represents a recurring mechanistic theme across the included studies, the immune microenvironment of chronic diabetic wounds is inherently multicellular and dynamically regulated. Beyond macrophages, immune dysregulation involving neutrophils and T lymphocytes contributes to the persistence of inflammation and impaired healing [74, 75]. Dysregulated neutrophil activity (including delayed clearance and excessive NET formation), impaired antigen‐presenting cell function, and altered T‐cell–mediated responses can shape chronic inflammation and tissue remodelling [76, 77]. In addition, innate immune pattern‐recognition programmes (e.g., TLR signalling) and inflammasome activation, together with metabolic stressors such as hyperglycaemia, oxidative stress/AGEs, and hypoxia, can interact to perpetuate non‐healing phenotypes [78, 79]. Notably, evidence published after our search window suggests that M2 macrophage‐derived extracellular vesicles functionalised within an ADM could enhance immunoregulatory signalling and angiogenesis in diabetic wound healing [80]. Therefore, future biomaterial and tissue‐engineering strategies should be designed and evaluated with a broader immune‐systems lens, integrating multi‐cell immune readouts and, where possible, human‐relevant models to improve translational interpretability [74, 76].

In addition to macrophage‐centred mechanisms, other immune contributors are increasingly recognised in diabetic ulcer chronicity. Persistent neutrophil infiltration and excessive NET formation can prolong inflammation and impair re‐epithelialisation and angiogenesis, while altered T‐cell responses further contribute to dysregulated repair. Moreover, neuroimmune crosstalk—particularly through sympathetic/adrenergic signalling—can shape inflammatory tone and tissue repair, as neurotransmitters and neuropeptides (e.g., norepinephrine, NPY, VIP, acetylcholine) modulate leukocyte behaviour, cytokine production, perfusion, and epithelial responses. Collectively, these layers underscore that DFU inflammation is regulated by a broader immune–neural network, supporting the need for future biomaterial and tissue‐engineering studies to report multi‐immune‐cell outcomes and, where feasible, neuroimmune/adrenergic readouts to improve translational relevance [74, 75, 76, 81, 82, 83].

In parallel with biomaterial‐based approaches, pharmacologic and repurposed therapies can also modulate chronic inflammation and have shown clinically relevant immunomodulatory effects and potential benefits in DFU management (e.g., anti‐inflammatory and pro‐resolving strategies, topical/systemic agents targeting dysregulated cytokine signalling, and drug‐based adjuncts used alongside standard wound care) [13]. Although drug‐based therapies were not the primary focus of the present biomaterial‐centred synthesis, they represent an important complementary arm of immunomodulation and are increasingly discussed in recent DFU pharmacotherapy and clinical‐trial overviews [84]. One clinically relevant example is topical timolol (a nonselective beta‐adrenergic antagonist), which has been used as an adjunctive therapy for chronic wounds and has been explored in DFUs. Proposed mechanisms include modulation of beta‐adrenergic stress signalling with downstream effects on inflammatory signalling and re‐epithelialisation/keratinocyte migration [85], and recent reviews and clinical reports suggest potential benefit and indicate that topical use is generally well tolerated [86, 87]. Future translational work may benefit from integrated regimens that combine immuno‐instructive biomaterials with well‐characterised pharmacologic immunomodulators to improve clinical relevance and scalability.

Emerging evidence suggests that immune dysregulation in DFU is also shaped by microbiome–immune interactions. Shifts in wound microbial communities and biofilm formation can sustain inflammatory signalling, impair barrier function, and influence healing trajectories, supporting the rationale for strategies that integrate antimicrobial/biofilm‐aware approaches with immunomodulation [88, 89]. In parallel, neurovascular–immune coupling is increasingly recognised as a determinant of chronicity: neuropathy and microvascular dysfunction alter perfusion, sensory signalling, and local immune tone, while sympathetic/adrenergic mediators can modulate inflammatory cell behaviour and epithelial repair responses [82, 90].

Beyond local biomaterials, nutraceutical and metabolic immunomodulators may influence wound immunometabolism and inflammatory resolution (e.g., via redox balance, micronutrient‐dependent immune functions, and metabolic reprogramming pathways), although standardised clinical evidence remains heterogeneous [91, 92].

Finally, commonly used systemic medications may exert pleiotropic immune and vascular effects relevant to DFU outcomes; for example, recent reviews discuss how GLP‐1 receptor agonists may modulate inflammation, microvascular function, and repair processes in the DFU context, underscoring the value of future integrative studies that combine systemic optimisation with local immuno‐instructive therapies [93, 94].

Although many included studies demonstrate accelerated closure and favourable early immunologic shifts, the current evidence base provides limited insight into whether these modalities deliver *durable* healing. In preclinical models, follow‐up is often short, and outcomes commonly emphasise closure kinetics and early inflammatory markers rather than post‐closure function and resilience. Clinically, DFU “healing” may be better conceptualised as *remission*, given that re‐ulceration is common after apparent closure. Therefore, future translational studies should extend follow‐up beyond early closure and report standardised durability‐oriented endpoints, including quality of regenerated tissue (barrier integrity, collagen architecture, vascular maturation), infection resistance/biofilm control, and post‐closure surveillance for recurrence and functional outcomes [95, 96].

To improve cross‐study comparability, future DFU immunomodulation studies should adopt harmonised immune outcome definitions and a pragmatic minimum reporting set. At a minimum, we recommend reporting a core cytokine panel (e.g., TNF‐α, IL‐1β, IL‐6, IL‐10, TGF‐β), standardised macrophage markers at consistent timepoints (e.g., CD68 plus paired M1/M2‐associated markers such as iNOS/CD86 and Arg1/CD206), and—where feasible—expanded readouts beyond macrophages (e.g., neutrophil and T‐cell endpoints). Harmonising these immune measures alongside clinically meaningful outcomes (healing time, infection, recurrence) would strengthen mechanistic conclusions and help identify the most effective immunomodulatory strategies [97].

Overall, these strategies have the potential to shorten healing time by simultaneously addressing chronic inflammation and insufficient vascularisation in DFU. However, the scarcity of human studies [35, 51] and limited long‐term safety data remain major barriers to clinical implementation.

### 5.1 Limitations

The vast majority of the 34 included studies relied primarily on preclinical animal models, which limits the generalizability of findings to the human DFU population. Substantial variability in animal models, cell types, biomaterial compositions, and immunomodulatory strategies contributed to heterogeneity and precluded robust comparative analyses or formal meta‐analyses. Moreover, most studies focused on short‐term wound closure and early regenerative outcomes, with limited assessment of long‐term tissue function, the quality of re‐epithelialisation, or DFU recurrence risk.

Across studies, inconsistency in the markers and quantitative metrics used to assess macrophage polarisation and angiogenesis further complicated cross‐study comparisons. In addition, the evidence base remains largely macrophage‐centric: many studies emphasised M1/M2 panels and rarely incorporated broader immune profiling (e.g., neutrophil persistence/NET‐related endpoints, T‐cell dysfunction, or neuroimmune/adrenergic measures). This limits a holistic interpretation of immune dysregulation in DFU and underscores the need for standardised, multi‐cellular immune outcome reporting in future translational research.

Finally, the immunogenic effects and long‐term toxicity profiles of cell‐, exosome‐, or nanoparticle‐based strategies remain insufficiently characterised, representing a critical barrier to clinical translation. Because this review prioritised bioengineered and biomaterial‐based immunomodulatory interventions, pharmacologic and repurposed therapies may be underrepresented. In addition, other emerging immunomodulatory domains—such as microbiome–immune interactions, neurovascular–immune coupling, nutraceutical or metabolic modulators, and systemic medication effects—were not exhaustively synthesised due to scope and heterogeneity, and should be prioritised in future integrative studies. As a result, evidence remains sparse on durability‐focused endpoints—such as barrier restoration, infection risk after closure, and ulcer recurrence—which are critical to judging sustained clinical benefit in DFU care.

## 6 Conclusion

The findings of this systematic review provide a framework for safely and effectively translating current evidence into clinical practice, highlighting that tissue‐engineering–based immunomodulation should be prioritised in future DFU research. Across studies, macrophage polarisation from the pro‐inflammatory M1 to the pro‐regenerative M2 phenotype emerged as a fundamental mechanism of healing. Cytokine delivery, exosome‐ or conditioned‐medium approaches, antioxidant nano‐sensors, and electrical or mechanical stimulation consistently accelerated tissue repair by resolving chronic inflammation. Platforms such as natural polymer hydrogels, electrospun nanofibers, and microneedle systems reduced oxidative stress and inflammation while stimulating angiogenesis. Cell‐ and gene‐targeted strategies—including IL‐27, IL‐4/IL‐10/IL‐13 over‐expressing MSCs, and MSC‐derived exosomes—significantly enhanced DFU healing by restoring macrophage polarisation and immune balance. Furthermore, targeting key signalling pathways such as JAK/STAT, NF‐κB, HMGB1–RAGE, and HIF‐1α provides molecular entry points for developing biomaterial‐based therapies that reprogram macrophage behaviour, promote angiogenesis, and modulate the inflammatory microenvironment. Collectively, these insights demonstrate the potential of tissue‐engineering strategies to shorten recovery time by resolving chronic inflammation, reducing oxidative stress, and enhancing vascularisation in DFU.

Although preclinical findings are promising, the scarcity of human‐based studies underscores the need for well‐designed randomised controlled trials and multicentre studies with adequate sample sizes and long‐term follow‐up protocols to support clinical translation.

Future work should integrate macrophage‐polarisation pathways (e.g., JAK/STAT, NF‐κB) and oxidative‐stress regulators directly into biomaterial design to achieve combined therapeutic effects. Incorporating multi‐omic data with biomarker‐based patient stratification may enable development of personalised immunomodulatory treatment algorithms for DFU. Because heterogeneity in biomaterial compositions, animal models, and immunomodulatory strategies precluded a formal meta‐analysis in this review, meta‐analytic studies focused on homogeneous subgroups—such as IL‐4/IL‐10‐loaded hydrogels or redox‐active nanozyme platforms—are recommended to strengthen the evidence base. Finally, future translational frameworks should consider integrated regimens that combine immuno‐instructive biomaterials with complementary immunomodulatory adjuncts (e.g., repurposed pharmacologic agents) and emerging host–immune domains (e.g., microbiome‐immune and neurovascular‐immune coupling) to improve clinical relevance.

## Funding

The authors have nothing to report.

## Conflicts of Interest

The authors declare no conflicts of interest.

## Supporting information


**Figure S1:** Risk‐of‐bias assessment for included primary studies using the OHAT (Office of Health Assessment and Translation) tool. Each domain (D1–D10) is colour‐coded: green = low risk, yellow = unclear risk.
**Figure S2:** Summary plot of OHAT risk‐of‐bias ratings across all primary studies, showing the percentage of judgements at each risk level for each domain.
**Figure S3:** Risk‐of‐bias assessment included secondary studies using the OHAT tool. Each domain (D1–D10) is colour‐coded: green = low risk, yellow = unclear risk.
**Figure S4:** Summary plot of OHAT risk‐of‐bias ratings across all secondary studies.
**Figure S5:** Risk‐of‐bias assessment of animal studies using the SYRCLE tool. Each domain (D1–D10) is colour‐coded: green = low risk, yellow = unclear risk, blue = no information.
**Figure S6:** Summary plot of SYRCLE risk‐of‐bias ratings across all animal studies, showing the proportion of judgements at each risk level for each domain.

**Table S1:** Full database search strategies and records retrieved.

**Table S2:** Methodological quality and risk‐of‐bias assessment of the included studies.

## Data Availability

The data that supports the findings of this study are available in the Supporting Information of this article.
