# Supplementary material for: Immunomodulatory Tissue‐Engineering Strategies for Diabetic Foot Ulcer Management: A Systematic Review
Source: Wound Repair Regen. 2026 Mar 25;34(2):e70149. doi: 10.1111/wrr.70149 (PMC13014567; doi:10.1111/wrr.70149)

**Supplementary Figure S1.** Risk-of-bias assessment for included primary studies using the OHAT (Office of Health Assessment and Translation) tool. Each domain (D1–D10) is color-coded: green = low risk, yellow = unclear risk.


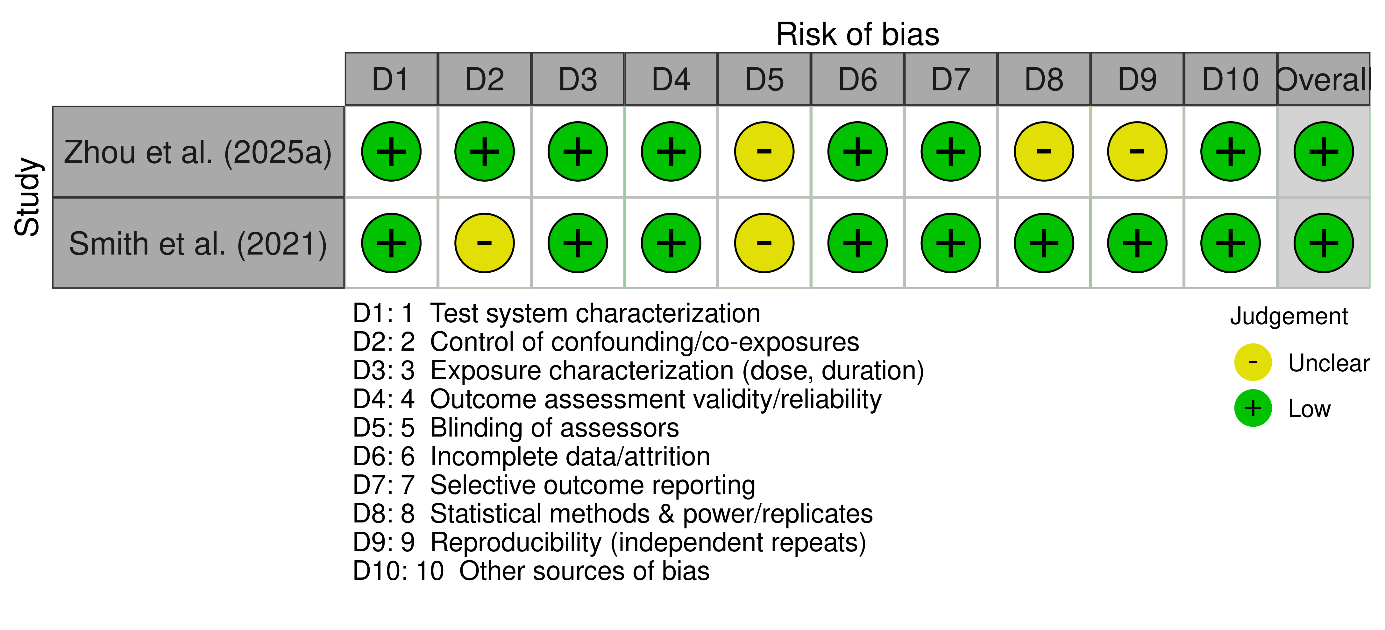


**Supplementary Figure S2.** Summary plot of OHAT risk-of-bias ratings across all primary studies, showing the percentage of judgments at each risk level for each domain.


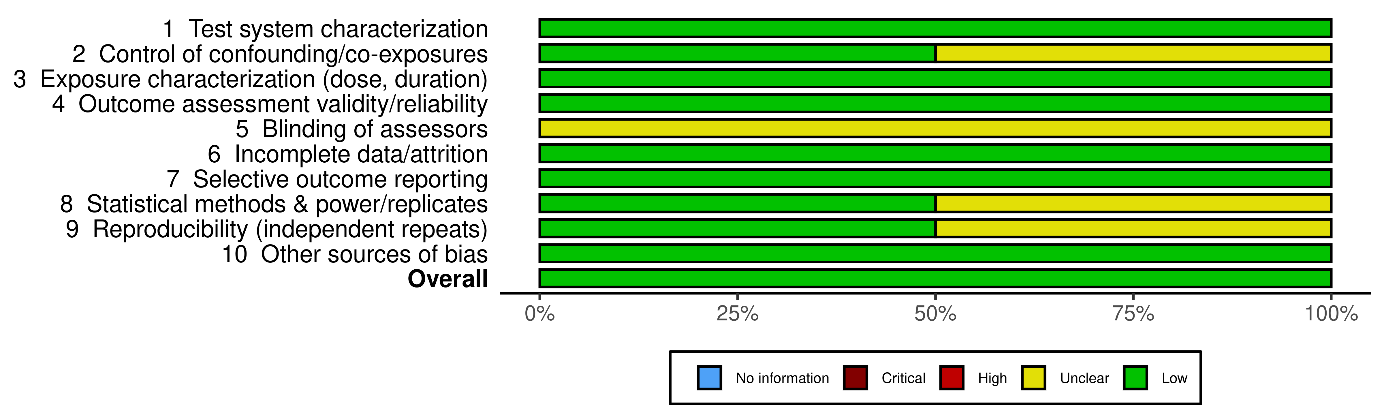


**Supplementary Figure S3.** Risk-of-bias assessment included secondary studies using the OHAT tool. Each domain (D1–D10) is color-coded: green = low risk, yellow = unclear risk.

**
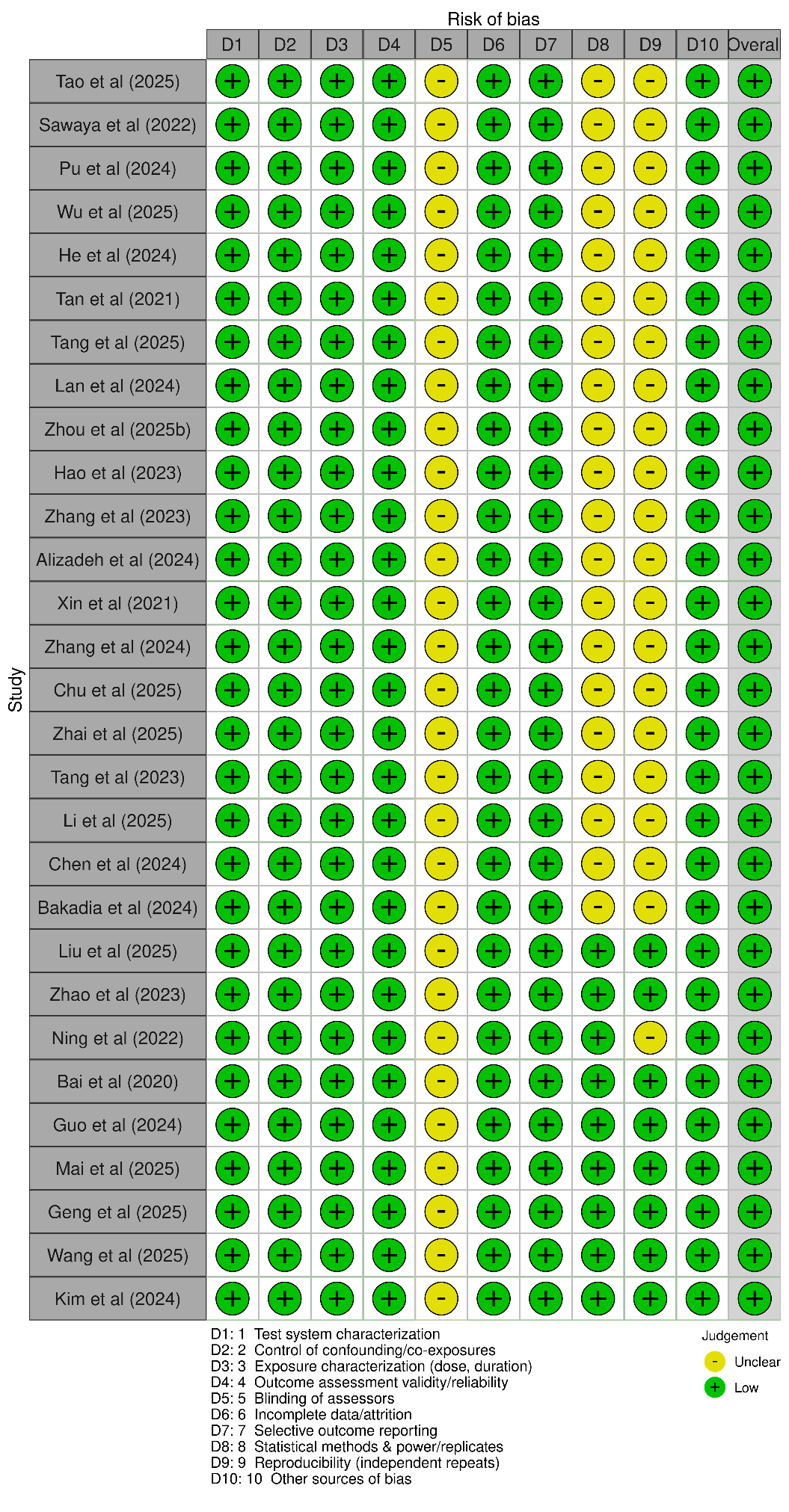
**

**Supplementary Figure S4.** Summary plot of OHAT risk-of-bias ratings across all secondary studies.


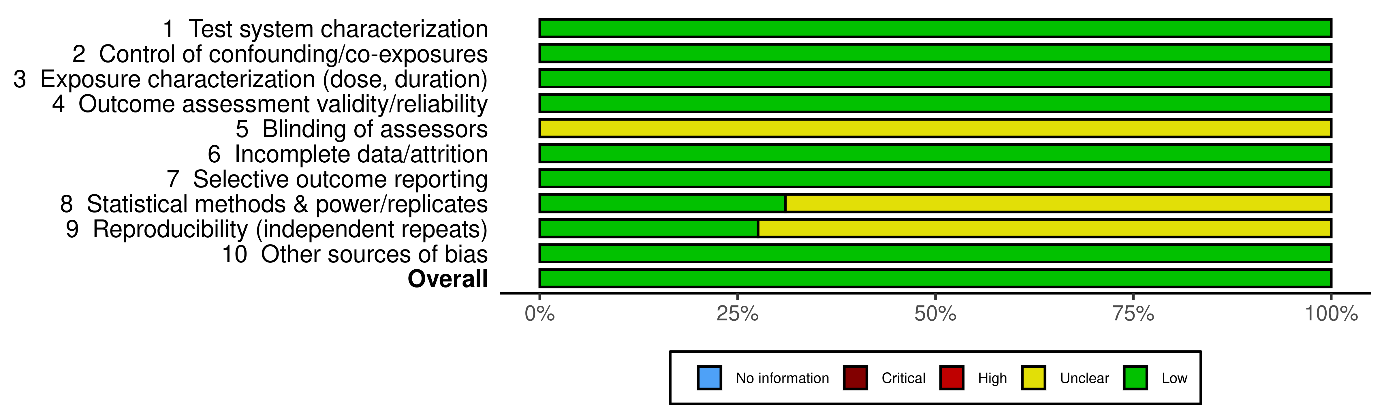


**Supplementary Figure S5.** Risk-of-bias assessment of animal studies using the SYRCLE tool. Each domain (D1–D10) is color-coded: green = low risk, yellow = unclear risk, blue = no information.


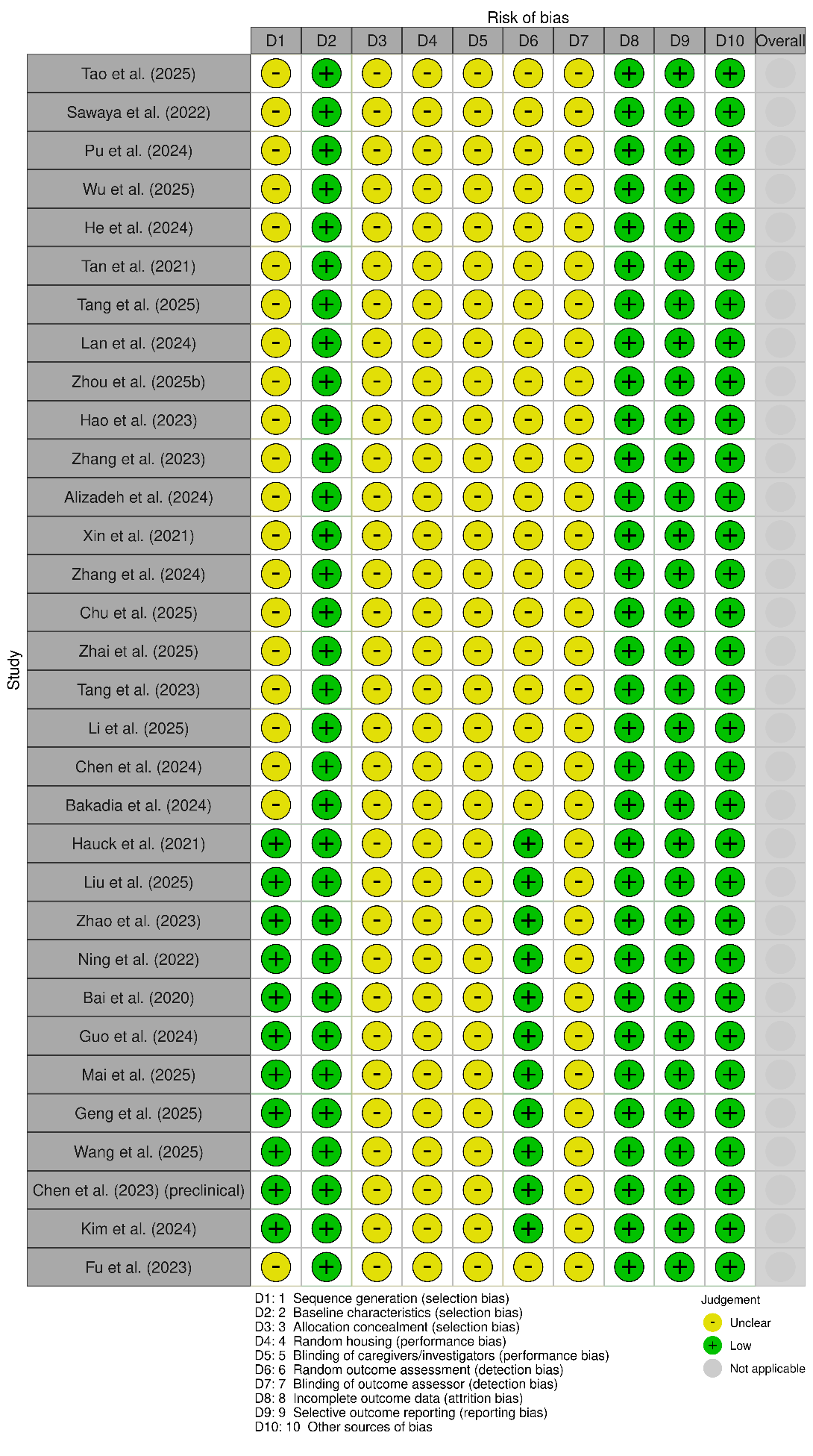


**Supplementary Figure S6.** Summary plot of SYRCLE risk-of-bias ratings across all animal studies, showing the proportion of judgments at each risk level for each domain.


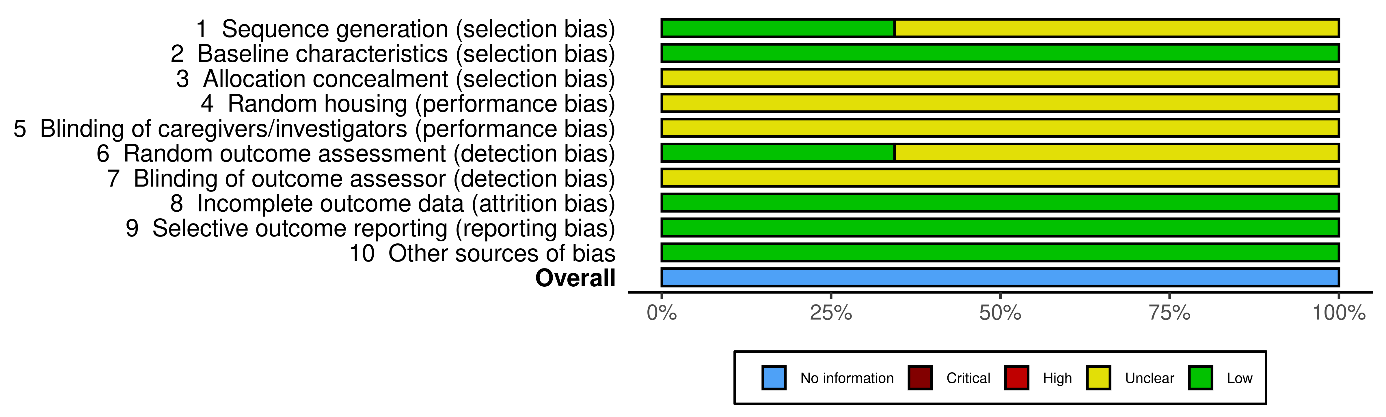

Supplement: Supplementary file 1 — Figure S1: Risk‐of‐bias assessment for included primary studies using the OHAT (Office of Health Assessment and Translation) tool. Each domain (D1–D10) is colour‐coded: green = low risk, yellow = unclear risk. Figure S2: Summary plot of OHAT risk‐of‐bias ratings across all primary studies, showing the percentage of judgements at each risk level for each domain. Figure S3: Risk‐of‐bias assessment included secondary studies using the OHAT tool. Each domain (D1–D10) is colour‐coded: green = low risk, yellow = unclear risk. Figure S4: Summary plot of OHAT risk‐of‐bias ratings across all secondary studies. Figure S5: Risk‐of‐bias assessment of animal studies using the SYRCLE tool. Each domain (D1–D10) is colour‐coded: green = low risk, yellow = unclear risk, blue = no information. Figure S6: Summary plot of SYRCLE risk‐of‐bias ratings across all animal studies, showing the proportion of judgements at each risk level for each domain. [file WRR-34-0-s002.docx]
