# Supplementary material for: Immunomodulatory Tissue‐Engineering Strategies for Diabetic Foot Ulcer Management: A Systematic Review
Source: Wound Repair Regen. 2026 Mar 25;34(2):e70149. doi: 10.1111/wrr.70149 (PMC13014567; doi:10.1111/wrr.70149)
Supplement: Supplementary file 2 — Table S1: Full database search strategies and records retrieved. [file WRR-34-0-s003.docx]

**Supplementary Table S1. Full database search strategies and records retrieved (final search: 14 September 2025)**

| **Focus area** | **Database** | **Full Boolean search string** | **Records retrieved** |
| --- | --- | --- | --- |
| **Diabetic Foot Ulcer + Tissue Engineering + Immunomodulation** | PubMed | ("Diabetic Foot"[Mesh] OR "diabetic foot ulcer" OR "diabetic wounds") AND ("Tissue Engineering"[Mesh] OR "Regenerative Medicine"[Mesh] OR "tissue engineering" OR "regenerative medicine") AND ("Immunomodulation"[Mesh] OR immunomodulation OR "immune response" OR "immune microenvironment") | 21 |
|  | Scopus | ("diabetic foot ulcer" OR "diabetic foot" OR "diabetic wounds") AND ("tissue engineering" OR "regenerative medicine") AND (immunomodulation OR "immune modulation" OR "immune response" OR "immune microenvironment") | 80 |
|  | Web of Science | TS=("diabetic foot ulcer" OR "diabetic foot" OR "diabetic wound") AND TS=("tissue engineering" OR "regenerative medicine") AND TS=(immunomodulation OR "immune modulation" OR "immune response" OR "immune microenvironment") | 5 |
| **Biomaterial and Scaffold Approaches** | PubMed | ("Diabetic Foot"[Mesh] OR "diabetic foot ulcer") AND (scaffold OR hydrogel OR biomaterials OR "3D printed scaffold" OR "nanofiber scaffold") AND (immunomodulation OR "immune response" OR "immune microenvironment") | 15 |
|  | Scopus | ("diabetic foot ulcer" OR "diabetic foot") AND (scaffold OR hydrogel OR biomaterials OR "3D printed scaffold" OR "nanofiber scaffold") AND (immunomodulation OR "immune response" OR "immune microenvironment") | 67 |
|  | Web of Science | TS=("diabetic foot ulcer" OR "diabetic foot") AND TS=(scaffold OR hydrogel OR biomaterials OR "3D printed scaffold" OR "nanofiber scaffold") AND TS=(immunomodulation OR "immune response" OR "immune microenvironment") | 8 |
| **Macrophage Polarization / Immune Mechanisms** | PubMed | ("Diabetic Foot"[Mesh] OR "diabetic foot ulcer") AND ("macrophage polarization" OR "M1 macrophage" OR "M2 macrophage") AND ("tissue engineering" OR "regenerative medicine") | 3 |
|  | Scopus | ("diabetic foot ulcer" OR "diabetic foot") AND ("macrophage polarization" OR "M1 macrophage" OR "M2 macrophage") AND ("tissue engineering" OR "regenerative medicine") | 13 |
|  | Web of Science | TS=("diabetic foot ulcer" OR "diabetic foot") AND TS=("macrophage polarization" OR "M1 macrophage" OR "M2 macrophage") AND TS=("tissue engineering" OR "regenerative medicine") | 3 |
| **Cell / Gene Therapies** | PubMed | ("Diabetic Foot"[Mesh] OR "diabetic foot ulcer") AND ("cell therapy" OR "stem cell therapy" OR "gene therapy") AND (immunomodulation OR "immune modulation") | 0 |
|  | Scopus | ("diabetic foot ulcer" OR "diabetic foot") AND ("cell therapy" OR "stem cell therapy" OR "gene therapy") AND (immunomodulation OR "immune modulation") | 24 |
|  | Web of Science | TS=("diabetic foot ulcer" OR "diabetic foot") AND TS=("cell therapy" OR "stem cell therapy" OR "gene therapy") AND TS=(immunomodulation OR "immune modulation") | 0 |
